# Interfacial solar vapor electrolyzer for efficient and durable hydrogen production directly from seawater

**DOI:** 10.1093/nsr/nwaf397

**Published:** 2025-09-18

**Authors:** Mengyue Zeng, Liyao Ji, Weichao Xu, Tianqi Wei, Miao Zhong, Zhaosheng Li, Ning Xu, Xing Zhang, Zhigang Zou, Jia Zhu

**Affiliations:** National Laboratory of Solid State Microstructures, College of Engineering and Applied Sciences, Jiangsu Key Laboratory of Artificial Functional Materials, Jiangsu Physical Science Research Center, Frontiers Science Center for Critical Earth Material Cycling, Collaborative Innovation Center of Advanced Microstructures, Nanjing University, Nanjing 210093, China; National Laboratory of Solid State Microstructures, College of Engineering and Applied Sciences, Jiangsu Key Laboratory of Artificial Functional Materials, Jiangsu Physical Science Research Center, Frontiers Science Center for Critical Earth Material Cycling, Collaborative Innovation Center of Advanced Microstructures, Nanjing University, Nanjing 210093, China; National Laboratory of Solid State Microstructures, College of Engineering and Applied Sciences, Jiangsu Key Laboratory of Artificial Functional Materials, Jiangsu Physical Science Research Center, Frontiers Science Center for Critical Earth Material Cycling, Collaborative Innovation Center of Advanced Microstructures, Nanjing University, Nanjing 210093, China; National Laboratory of Solid State Microstructures, College of Engineering and Applied Sciences, Jiangsu Key Laboratory of Artificial Functional Materials, Jiangsu Physical Science Research Center, Frontiers Science Center for Critical Earth Material Cycling, Collaborative Innovation Center of Advanced Microstructures, Nanjing University, Nanjing 210093, China; National Laboratory of Solid State Microstructures, College of Engineering and Applied Sciences, Jiangsu Key Laboratory of Artificial Functional Materials, Jiangsu Physical Science Research Center, Frontiers Science Center for Critical Earth Material Cycling, Collaborative Innovation Center of Advanced Microstructures, Nanjing University, Nanjing 210093, China; National Laboratory of Solid State Microstructures, College of Engineering and Applied Sciences, Jiangsu Key Laboratory of Artificial Functional Materials, Jiangsu Physical Science Research Center, Frontiers Science Center for Critical Earth Material Cycling, Collaborative Innovation Center of Advanced Microstructures, Nanjing University, Nanjing 210093, China; National Laboratory of Solid State Microstructures, College of Engineering and Applied Sciences, Jiangsu Key Laboratory of Artificial Functional Materials, Jiangsu Physical Science Research Center, Frontiers Science Center for Critical Earth Material Cycling, Collaborative Innovation Center of Advanced Microstructures, Nanjing University, Nanjing 210093, China; National Laboratory of Solid State Microstructures, College of Engineering and Applied Sciences, Jiangsu Key Laboratory of Artificial Functional Materials, Jiangsu Physical Science Research Center, Frontiers Science Center for Critical Earth Material Cycling, Collaborative Innovation Center of Advanced Microstructures, Nanjing University, Nanjing 210093, China; National Laboratory of Solid State Microstructures, College of Engineering and Applied Sciences, Jiangsu Key Laboratory of Artificial Functional Materials, Jiangsu Physical Science Research Center, Frontiers Science Center for Critical Earth Material Cycling, Collaborative Innovation Center of Advanced Microstructures, Nanjing University, Nanjing 210093, China; National Laboratory of Solid State Microstructures, College of Engineering and Applied Sciences, Jiangsu Key Laboratory of Artificial Functional Materials, Jiangsu Physical Science Research Center, Frontiers Science Center for Critical Earth Material Cycling, Collaborative Innovation Center of Advanced Microstructures, Nanjing University, Nanjing 210093, China; School of Sustainable Energy and Resources, Nanjing University, Suzhou 215010, China

**Keywords:** interfacial solar evaporation, solar hydrogen production, seawater electrolysis, photovoltaic-electrolysis system

## Abstract

Solar-powered water splitting from seawater offers a sustainable pathway for hydrogen production, yet current methods are challenged by the complex composition of seawater, as well as intrinsic thermodynamic and kinetic limits when using liquid water as reactants. This work proposes an interfacial solar vapor electrolyzer (ISVE), which synergistically utilizes interfacial vapor generation driven by dissipated solar-thermal energy and solar-powered electricity to enable efficient and stable hydrogen (H_2_) production directly from seawater. Abundant solar-thermal vapor as the reactant during electrolysis not only significantly enhances durability due to intrinsically purified properties, but also fundamentally increases the solar-to-hydrogen (STH) efficiency owing to reduced thermodynamic and kinetic barriers. As a demonstration, ISVE achieves a 15.2% STH efficiency at an average electrolysis current density of over 140 mA cm^−^^2^, and also exhibits stable operation for over 1400 h with seawater. This design enables efficient and durable solar H_2_ production directly from widely available water sources, offering a cost-effective solution with high potential for practical applications.

## Teaser

A solar vapor electrolyzer that synergistically utilizes solar electricity and solar thermal vapor was developed for efficient and stable hydrogen production, providing a promising pathway to sustainable clean energy solutions.

## INTRODUCTION

Solar-driven hydrogen (H_2_) production, which converts the most abundant renewable energy source into green hydrogen, has emerged as a promising technology for global energy decarbonization and sustainable development [1–3]. Current water electrolysis technologies typically rely on freshwater, a scarce commodity worldwide, thereby limiting their scalability [4]. Seawater, constituting over 96% of water resources on Earth, offers a viable alternative [5]. Despite its vast availability, seawater electrolysis faces significant challenges primarily due to its complex chemical composition [6–8]. Impurities such as chloride ions and alkaline earth metal ions will cause electrode corrosion and membrane fouling, thus leading to serious performance degradation [9,10]. Various innovative strategies [11–16], such as catalyst engineering and pre-purification processes, have been employed to address these issues. However, significant efforts are needed to further decrease system complexity and improve economic viability [15,17,18].

Recent advancements in interfacial solar evaporation offer new opportunities [19–23]. This technology efficiently captures sunlight and localizes the photothermal heat at the air–water surface to generate high-flux purified vapor while naturally excluding salts and contaminants [24–29]. Although this technology still faces challenges, such as achieving consistent high-flux vapor generation under fluctuating environmental conditions, ensuring long-term stability in real seawater and scaling up to commercial deployment, its rapid development and expanding applications make it play a critical role in addressing global water scarcity and the growing demand for clean energy [25,30]. Intriguingly, vapor-phase reactants also demonstrate two distinct advantages over liquid water: reduced Gibbs free energy requirements [31,32] and elimination of mass transfer limitations caused by bubble accumulation [32–35]. These findings have been experimentally validated, although through studies employing carrier-gas-mediated vapor delivery systems [32,33,35–37]. Interfacial solar evaporation offers a direct approach for producing high-flux purified vapor without the need for extra energy/apparatus inputs, which is predicted to be beneficial for electrolysis. Moreover, this solar vapor is typically at elevated temperatures, which is advantageous for enhancing the performance of water splitting. Thus, the synergetic integration of solar thermal vapor and solar electricity in one device for H_2_ generation is expected to be an ideal approach to generating green H_2_ with higher conversion efficiency and enhanced water source tolerance. Simplified quantitative analysis also proves the initial feasibility. Taking the efficiency of an ideal single-junction photovoltaic (PV) cell and considering a moderate osmotic drag coefficient (∼1) of electrochemical electrolyzer (EC) for water splitting [38], the maximum vapor flux required by a PV-EC system is calculated to be ∼0.27 kg h^−^^1^ for standard sunlight illumination (1 kW m^−^^2^), which can be conveniently met by the efficient interfacial evaporation driven by the solar-thermal energy [19,21,23,39].

Here, we propose an interfacial solar vapor electrolyzer (ISVE) for efficient and durable H_2_ production directly from seawater. The high-flux hot vapor generated via interfacial evaporation, driven by waste heat generated from the PV component, is directly utilized for solar-powered electrolysis. Therefore, ISVE enables significantly improved solar-to-hydrogen (STH) efficiency and durability compared to its liquid-phase counterpart fed with seawater, without extra energy or apparatus. As a demonstration, ISVE realizes a stable STH efficiency of 15.2% when feeding seawater, while the STH efficiency of the PV-driven seawater electrolyzer quickly drops under the same conditions. Theoretical analysis further suggests that the ISVE has the potential to achieve higher efficiency when well matched with more efficient PV cells. Moreover, the ISVE, which is fed by seawater, also operates stably for over 1400 h without obvious degradation. A real outdoor test of ISVE has proved its potential for practical applications.

## RESULTS

### Design of the integrated solar-driven vapor electrolyzer (ISVE)

**Figure 1.**
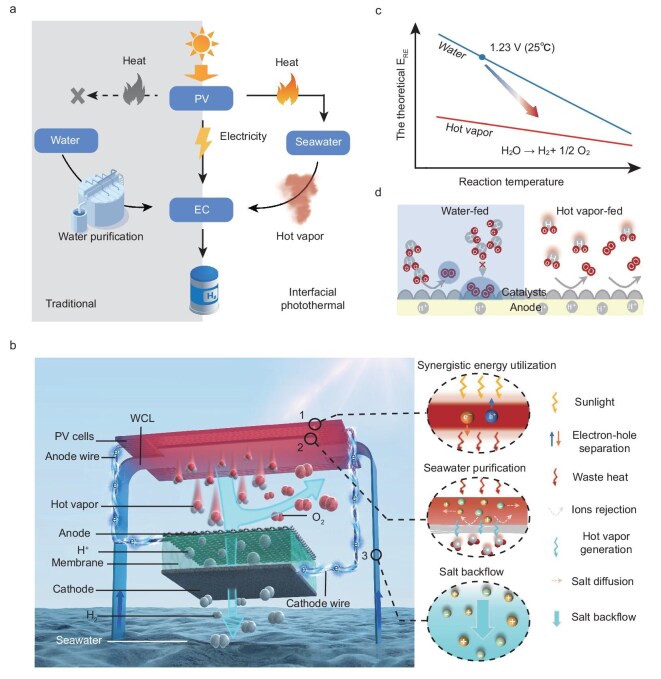
Design of ISVE. (a) The differences between the conventional PV-EC water splitting system (left) and ISVE system (right). (b) Schematic diagram of ISVE, which comprises a PV cell, a WCL and a PEM electrolyzer from top to bottom: (1) the top PV cell absorbs sunlight to generate electricity for electrolysis and produces waste heat for interfacial evaporation; (2) seawater is vaporized into hot vapor, which passes through the gas path, while solutes are retained and diffuse along the concentration gradient; (3) salt ions diffuse back into the bulk seawater, ensuring continuous operation. (c) Theoretical E_RE_ for water splitting as a function of temperature, comparing hot vapor-fed and water-fed electrolysis. (d) Schematic illustration of the adverse effect of gas-product bubble accumulation on the accessibility of the active sites of the anode when fed with liquid water as a reactant (left). Differently, bubble blocking is absent in hot vapor-fed electrolysis (right).

The key feature of ISVE lies in its utilization of purified hot vapor as the reactant, which is generated *in situ* from solar-derived heat through interfacial evaporation (Fig. 1a, Fig. S1). Unlike traditional PV-EC systems fed by seawater, ISVE eliminates the need for additional energy or apparatus. The design of the ISVE device is shown in Fig. 1b. The system, from top to bottom, mainly consists of a PV cell, an elaborately designed water-supply and contamination-preventive layer (WCL), and a proton-exchange-membrane (PEM) electrolyzer for vapor electrolysis. The PV cell converts sunlight into electricity with high solar-to-electric efficiency while simultaneously generating waste heat (Fig. 1b 1). Unlike conventional systems, where waste heat is a burden, the ISVE *in situ* reutilizes this heat to drive the interfacial evaporation process in the WCL. Within the WCL, seawater is transported via capillary action and undergoes a thermally driven phase transition into hot vapor, serving as the reactant for electrolysis, while detrimental ions are prevented from damaging the EC component (Fig. 1b 2). Furthermore, the excessive salt can flow back into the bulk water through the brine-filled microchannels of the WCL via diffusion and convection, ensuring continuous operation (Fig. 1b 2 and 3).

Employing hot vapor as the feedstock provides multiple advantages over seawater. In terms of the purity of water, the *in situ*-purified hot vapor mitigates interference from salt ions, thereby ensuring the long-term stability of the system. Thermodynamically, hot vapor reduces the Gibbs free energy required for water splitting, lowing the theoretical thermodynamic reversible voltage (E_RE_) compared to liquid-phase reactants [31,32] (Fig. 1c, Fig. S2). Kinetically, the solid−gas reaction interface enables rapid desorption of gaseous products, preventing bubble accumulation at liquid-filled interfaces [32–34,40], thereby significantly boosting mass transfer for water-splitting reactions (Fig. 1d). Thus, this innovative approach can improve solar energy utilization and enable direct seawater electrolysis with increased STH efficiency and durability.

### Advantages of vapor electrolysis

**Figure 2.**
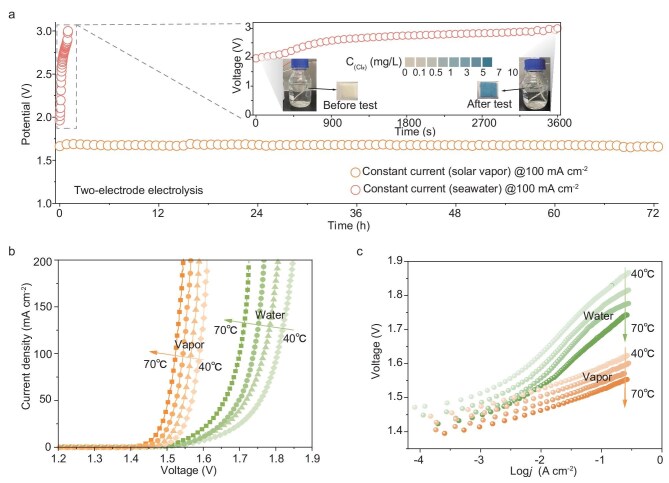
The advantages of vapor electrolysis. (a) Durability tests of vapor and seawater electrolysis at constant current densities of 100 mA cm^−2^. The inset shows the durability test of the PV-EC system with direct seawater feed for 1 h, along with the variation of chloride concentration in the feed seawater before and after the test. (b) The *iR*-corrected polarization curves of electrolyzers fed with either water or vapor under varied temperatures. (c) Temperature-dependent Tafel plots of electrolyzers fed with either water or solar vapor.

To validate the feasibility of utilizing interfacial photothermal vapor as a reactant, we implemented solar vapor generated from seawater at 50°C, which was directly introduced into a homemade PEM electrolyzer (see details in methods, Figs S3–S7). As shown in Fig. 2a, the system maintains stable operation over 72 h of continuous vapor electrolysis at a current density of 100 mA cm⁻^2^. In contrast, direct electrolysis of seawater at room temperature with the conventional PV-EC system exhibits a rapid voltage increase due to the high chloride ion concentrations in the feed seawater and membrane fouling [41]. These results suggest that the utilization of solar vapor as the reactant is promising for efficient and sustainable H_2_ generation through water electrolysis.

Building upon these results, we further evaluated the performance of H_2_ production via solar vapor electrolysis compared to liquid water electrolysis across various temperatures (Fig. S7). Theoretically, gaseous water electrolysis exhibits a 44 mV reduction of E_RE_ compared to liquid water under standard conditions [32]. Experimentally, the vapor-fed system demonstrated significantly lower onset voltages of water splitting than those of the liquid water-fed counterpart at equivalent temperatures (Fig. 2b). Kinetically, unlike liquid-phase systems, where limited oxygen solubility induces bubble accumulation and obstructs active sites, the gas–solid interface in vapor-fed electrolysis enables rapid O_2_ desorption, ensuring efficient catalytic turnover. This enhanced mass transport of solar vapor is further evidenced by a linear Tafel plot over a broad current density range, confirming kinetic control, whereas liquid water electrolysis shifts to both kinetics and mass-transfer joint control at lower current densities (Fig. 2c, Fig. S8). Moreover, increasing the temperature further reduces operating voltages for both systems by lowering the thermodynamic barrier for water splitting and accelerating reaction kinetics, as indicated by diminishing Tafel slopes and applied voltages at various current densities (Fig. 2c, Figs S8 and S9). Notably, the performance advantage of vapor over liquid water is also observed when using a more cost-effective RuOx catalyst at different temperatures (Fig. S10), further demonstrating the practical potential of using vapor as the reactant in cost-effective electrolysis systems. Additionally, the Faradaic efficiency of the EC component with solar vapor from seawater under different current densities is nearly 100% (Fig. S11). Thus, the ISVE enables durable and stable operation without catalyst deactivation, device corrosion and membrane blocking.

### Construction of the ISVE

**Figure 3.**
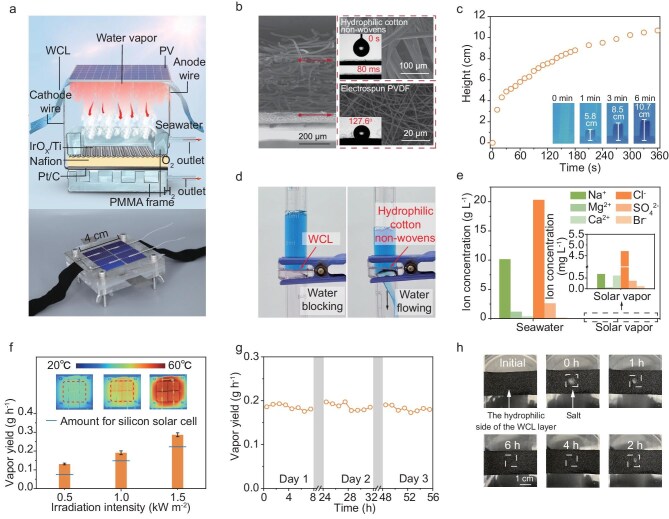
Construction of the ISVE. (a) Schematic and physical photos of the ISVE. (b) Scanning electron microscopy (SEM) image of the side view of the WCL (scale bar, 200 μm), consisting of hydrophilic cotton non-wovens (scale bar, 100 μm) and electrospun PVDF (scale bar, 20 μm). The inserts are the contact angles of the two sides. (c) Anti-gravity transport of water along the WCL. The inserts show infrared images of the water supply process. (d) Visual experiment comparison to demonstrate the waterproof characteristics of the WCL and the hydrophilic cotton non-wovens. (e) Concentrations of the primary ions of pristine seawater and interfacial solar vapor generated in the ISVE. (f) Temperature profile and vapor yield of the ISVE with a silicon PV cell under different solar illumination with *F* = ^1^/_16_. The required vapor amounts for the silicon PV cell are shown as blue lines. The inset displays infrared images of the interfacial evaporation system. Error bars represent standard errors. (g) Vapor production from seawater by the ISVE for three consecutive days. Each day involved 8 h of evaporation under illumination, generating purified vapor, followed by 16 h of darkness for salt backflow. (h) Optical photos illustrating the salt ion diffusion backflow process within the WCL (scale bar, 1 cm).

Here, as a demonstration, we used a silicon-based PV cell, a well-designed WCL and a PEM electrolyzer to construct the ISVE. The schematic and physical images of the ISVE are depicted in Fig. 3a, with additional details shown in Figs S12 and S13. The WCL plays a critical role in ISVE. First, it enables a sufficient water supply. Second, it utilizes the dissipated heat of PV for efficient hot vapor generation. Third, it allows vapor to pass through effectively while preventing contamination caused by seawater leakage. Finally, it allows the salt to diffuse back into the bulk seawater.

To construct the WCL, we deposited poly(vinylidene fluoride) (PVDF) nanofibers on the hydrophilic cotton non-woven substrate by electrospinning (Fig. 3b, see details in the Methods). The hydrophilic cotton non-woven substrate has good wettability, which can absorb droplets in less than 80 ms, enabling rapid water supply via capillary force (Fig. 3c). Also, WCL is designed to be thin (∼400 μm) for efficiently utilizing the dissipated heat of PV to generate sufficient hot vapor from the seawater via interfacial evaporation. The PVDF side of the WCL is porous and hydrophobic, which enables efficient vapor permeability while preventing seawater dripping (Fig. 3d). As a result, the ion concentrations of harmful ions (Na^+^, Mg^2+^, Cl^−^, etc.) in generated vapor through the WCL significantly decreased by orders of magnitude compared to those in the pristine seawater collected from the Bohai Sea (Fig. 3e). The ion concentration was determined by an inductively coupled plasma optical emission spectrometer (ICP-OES, PerkinElmer Avio 200). Meanwhile, the accumulated ions can diffuse and return to the bulk seawater through the hydrophilic layer of the WCL.

It should be noted that the ratio of electrolyzer/evaporation area to PV cell area (defined as the parameter *F*) is another critical parameter, reflecting the concentration degree of electricity and PV heat for electrolysis and vapor generation, which governs system performance and cost. Decreasing *F* results in more concentrated electrical output and PV-derived heat on a smaller active area, which not only increases electrolysis current density and reduces the required electrolyzer size to lower the construction cost (Note S1, Figs S14 and S15), but also elevates vapor temperature to improve efficiency (Fig. S16). However, as *F* is excessively reduced, corresponding to the overly high concentration degree, thermal losses become comparatively significant, causing the thermal vapor yield to fall below the vapor demand (Fig. S17).

To balance vapor yield supply and electrolysis efficiency while minimizing the required electrolyzer area to reduce the overall construction costs for each ISVE (Note S2), the system is elaborately designed with an *F* value of ^1^/_16_. At this configuration, the ISVE coupled with the silicon PV cell generates sufficient vapor yield across different illumination intensities (Fig. 3f, Fig. S18, Note S3). Furthermore, the generated vapor consistently exceeds room temperature (25°C), which benefits water splitting in terms of both thermodynamics and kinetics. Also, the ISVE system demonstrates stable evaporation performance with seawater over three consecutive days under an 8-h light and 16-h dark cycle (Fig. 3g), owing to the abundant hydrophilic microchannels of the WCL that facilitate water transport, salt diffusion and convection. This can be verified by the salt-adding experiment: approximately 0.05 g of salt, equivalent to the salt produced by the water required for 1 day of ISVE operation under 1 sun (considering an osmotic drag coefficient of ∼1) with Bohai seawater (∼3 wt%), can diffuse back to the bulk water via the WCL within 6 h (Fig. 3h), ensuring continuous operation.

### Performance of the ISVE

**Figure 4.**
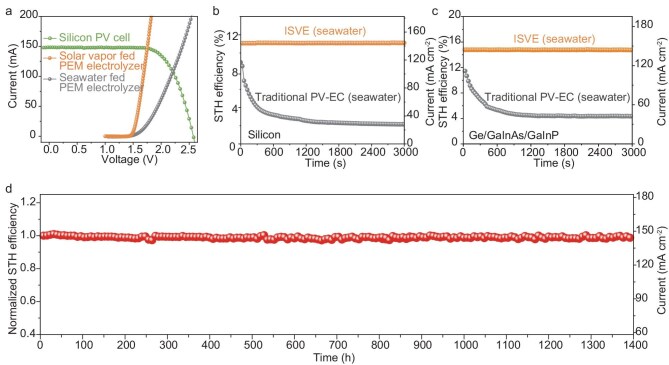
Performance of the ISVE indoor. (a) *J*–*V* curves of the silicon PV cell (16 cm^2^) under simulated 1-sun illumination (1 kW m^-2^) and the recorded initial polarization curves of the solar vapor and seawater fed PEM electrolyzers (effective area: 1 cm^2^). (b and c) STH efficiencies and current densities of ISVE and PV-EC systems coupled with a silicon PV cell (b) or a Ge/GaInAs/GaInp PV cell (c) under simulated 1-sun illumination when directly feeding seawater. (d) Long-term operation durability of the ISVE under 1-sun illumination. The performance metrics of the ISVE are shown in the form of normalized STH efficiency and current density.

By integrating with the WCL for interfacial evaporation, the ISVE, utilizing *in situ*-generated hot and pure vapor by dissipated heat of PV, is capable of realizing much better performance in solar H_2_ production compared to a PV-driven seawater electrolyzer. The silicon PV cell (16 cm^−2^) with a solar-to-electric power conversion efficiency (PCE) of 16.9% is used for the test (Fig. S19 and Table S1). The initial polarization curves of the traditional PV-EC electrolyzer and ISVE fed with seawater are illustrated in Fig. 4a, and the operating point of the systems under 1-sun illumination occurs at the intersection of the polarization curve of the electrolyzer and PV cell. The performance of the ISVE and the PV-EC system with seawater feed coupled with the silicon PV cell is further evaluated (Fig. 4b). With seawater spontaneously turning to purified hot vapor driven by the waste heat of PV, the ISVE delivers an outstanding average STH efficiency of 11.1% (silicon) at the water splitting current density of ∼144 mA cm^−2^. However, the PV-EC system suffered a severely reduced STH efficiency due to the complex composition of the seawater, which caused significant membrane fouling, side reaction and corrosion/dissolution of the anodic oxygen evolution catalyst (Figs. S20–S22). Performance degradation is also observed in the EC component with direct seawater feed (Fig. S23). Importantly, our design is widely compatible with various PV components, and thus a higher STH efficiency is expected to be achieved with a more efficient PV cell. When a high-efficiency Ge/GaInAs/GaInp (12 cm^2^) solar cell (PCE = 26.6%) was used, the ISVE achieved an STH efficiency of 15.2% at a water splitting current density of ∼148 mA cm^−^^2^ with excellent operation durability (Fig. 4c, Fig. S24), standing out from previous work of solar seawater splitting (Tables S6 and S7).

The ISVE has demonstrated excellent operational stability over 1400 h for solar H_2_ production directly from seawater (Fig. 4d). This is attributed to three main factors. Firstly, the *in situ*-purified vapor for catalysis avoids impurities that may reduce the performance of both catalysts and the membrane. Secondly, different from liquid water flow, the vapor as the reactant will not damage the mechanical robustness of the catalyst electrode [42]. Finally, concentrated salt ions during evaporation can diffuse back along the WCL effectively to prevent salt precipitation at the evaporation area. Such sustainable and efficient H_2_ production directly from seawater confirms the great potential of our ISVE for practical applications.

### Practicability of ISVE when directly fed with seawater

**Figure 5.**
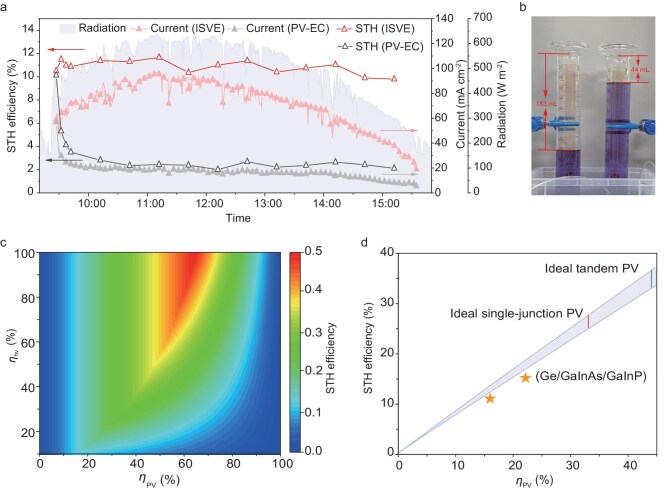
The practicability of ISVE. (a) Electrolysis current densities and STH efficiencies of ISVE and PV-EC devices with seawater feed driven by outdoor natural sunlight. (b) H_2_ production from direct seawater feed ISVE (left) and traditional PV-EC system (right) collected by the drainage method. (c) Simulation of the STH efficiency of ISVE over the PV efficiency (*η*_PV_) range of 0%–100% with *η*_hv_ varying from 10% to 100%. The temperature was 50°C and *F* = ^1^/_16_. (d) Theoretical STH efficiencies of ISVE (purple) varied with *η*_PV_. The temperature ranged from 40°C to 70°C, and the *F* ranged from ^1^/_25_ to 1. The *η*_hv_ was 50%. The STH efficiencies of ISVE with ideal single-junction and tandem PV, along with the measured performance achieved in this work using ISVE with silicon and Ge/GaInAs/GaInP PV cells, are also depicted.

To further demonstrate the practicability under real application scenarios, we conducted outdoor experiments for ISVE and conventional PV-EC systems on the campus of Nanjing University. A silicon-based PV component was utilized, and seawater was fed as the reactant (Fig. S25). During the test, ambient temperature, humidity and solar irradiance varied from 9°C to 16°C, 25% to 69%, and 180 to 640 W m⁻², respectively (Fig. S26). The performance metrics of the two systems are shown in Fig. 5a in the form of STH efficiency and overall current. Despite fluctuations in environmental conditions, the ISVE maintained a steady STH efficiency of ∼11% throughout the day, demonstrating its potential for reliable operation under practical scenarios. In contrast, the seawater-fed PV-EC electrolyzer showed a rapid decrease in STH efficiency, from 10.1% to 4.1% in the first 10 min, similar to the indoor experiment results. After the whole day’s test, the average current output for the seawater-fed PV-EC system was only 16.5 mA cm^−2^, corresponding to an STH efficiency of 2.5%. As quantified by the drainage method, the total H_2_ yield of ISVE was 183 mL, which is 4.3 times that of traditional PV-EC (Fig. 5b).

The potential of the ISVE system with seawater feed was further evaluated by developing a model to estimate the STH efficiency of ISVE when coupled with PV components of varying efficiencies. Unlike traditional PV-EC systems, which solely utilize solar energy for electrolysis, the ISVE system harnesses solar energy not only for electrolysis but also for generating heat to produce purified hot vapor from seawater. Thus, the STH efficiency of the ISVE is governed by two critical factors: the PV efficiency (*η*_PV_) and the heat-to-vapor efficiency (*η*_hv_). We conducted calculations of STH efficiency across a range of *η*_PV_ and *η*_hv_ values, with the detailed calculations provided in Note S4. As depicted in Fig. 5c, the performance of the ISVE is closely associated with the *η*_PV_ and *η*_hv_. When the *η*_PV_ is below 15% and *η*_hv_ is 10%, the STH efficiency of ISVE increases with *η*_PV_, as the generated solar vapor is sufficient for electrolysis. However, when the *η*_PV_ exceeds 15% and *η*_hv_ is ≤50%, there is a trade-off between solar energy for electricity generation and vapor generation. In this case, both *η*_PV_ and *η*_hv_ jointly determine the efficiency of the system. With *η*_hv_ is ≥50% and *η*_PV_ is ≤50%, the water vapor production is sufficient for electrolysis, thus, the *η*_PV_ dominates the performance of the system. A more detailed analysis (Fig. S27) further shows that as *η*_hv_ increases, the maximum attainable STH efficiency shifts toward higher *η*_PV_ values, demonstrating the importance of effective thermal management and optimized PV-EC coupling. So far, the *η*_hv_ in solar evaporation systems for water and electricity cogeneration has been reported as over 50% with good thermal management (Table S8), which means that with elaborate device design, the generated solar vapor is sufficient to support the ISVE system for H_2_ production in real-world applications, given that most PV cells have *η*_PV_ of ≤50%. Specifically, we employed an *η*_hv_ value of 50% and varied *η*_PV_ from 0% to 50% to show the potential of the ISVE system with direct seawater feed (Fig. 5d). The results demonstrate that the STH efficiency of ISVE, coupled with both ideal single-junction and tandem PV, can substantially surpass 20%, the target set by the U.S. Department of Energy (DOE) [43]. Figure 5d also includes the practically achieved STH efficiency of ISVE with silicon and Ge/GaInAs/GaInp PV cells. The slight discrepancy between the experimental and theoretical values is due to the efficiency loss from the integrated system impedance in the practical ISVE setup.

## DISCUSSION

We designed an ISVE device by synergistically integrating the interfacial solar vapor generation and PV-powered electrolyzer, which can efficiently and stably generate green H_2_ directly from polluted open water sources. Compared to conventional liquid-phase seawater splitting, the ISVE exhibits more efficient solar H_2_ production performance, owing to the purified nature of the vapor, which eliminates the need for complex catalyst engineering or pre-purification of diverse feedstocks to prevent catalyst poisoning or side reactions. Additionally, vapor electrolysis exhibits lower thermodynamic and kinetic barriers than liquid water electrolysis. As a demonstration, the ISVE achieves an STH efficiency of 15.2% at a current density of 148 mA cm^−^^2^ under normal sunlight illumination. Also, simulation results demonstrate that the STH efficiency of the ISVE system can be further promoted beyond 30% by using more efficient and optimally matched PV-EC coupling. Moreover, the ISVE also demonstrates operational durability over 1400 h when directly fed with seawater. Therefore, this work offers a promising strategy to promote solar-driven water splitting efficiency and durability with the direct utilization of widespread natural water sources.

We expect that the STH efficiency and stability of the ISVE can be further enhanced by more scientifically rational management of photons, electrons, heat and water, enabled by advances in both materials innovation and device engineering. Key directions include the development of durable and cost-effective electrocatalysts and ion-exchange membranes tailored for vapor-fed conditions, along with the advancement of PV cells and interfacial evaporation materials to synchronously enhance power conversion efficiency and purified vapor generation. Robust system integration, including optimized PV-EC coupling and precise thermal regulation, will be critical to adapt to unavoidable fluctuations in solar irradiance and ambient conditions. In particular, enhanced thermal regulation ensures stable and efficient operation even in cold climates. In parallel, the establishment of H_2_ storage and transport infrastructure will be essential to support large-scale deployment. Together, these advancements will accelerate the transition of ISVE from laboratory-scale demonstrations to practical, real-world applications.

## Supplementary Material

nwaf397_Supplemental_File
