# Supplementary material for: Interfacial solar vapor electrolyzer for efficient and durable hydrogen production directly from seawater
Source: Natl Sci Rev. 2025 Sep 18;12(11):nwaf397. doi: 10.1093/nsr/nwaf397 (PMC12581894; doi:10.1093/nsr/nwaf397)
Supplement: nwaf397_Supplemental_File [file nwaf397_supplemental_file.pdf]

Supplementary Materials for

Interfacial solar vapor electrolyzer for efficient and durable hydrogen production directly from seawater

Mengyue Zeng[1,†], Liyao Ji[1,†], Weichao Xu[1,†], Tianqi Wei[1], Miao Zhong[1], Zhaosheng Li[1], Ning Xu[1,*], Xing Zhang[1,*], Zhigang Zou[1] and Jia Zhu[1,2,*]

[1]National Laboratory of Solid State Microstructures, College of Engineering and Applied Sciences, Jiangsu Key Laboratory of Artificial Functional Materials, Jiangsu Physical Science Research Center, Frontiers Science Center for Critical Earth Material Cycling, Collaborative Innovation Center of Advanced Microstructures, Nanjing University, Nanjing 210093, China;

[2]School of Sustainable Energy and Resources, Nanjing University, Suzhou 215010, China

***Corresponding authors**. E-mails: nxu@nju.edu.cn; zhangxing@nju.edu.cn; jiazhu@nju.edu.cn

[†]Equally contributed to this work.

Materials and Methods

Materials

The chemicals and materials used in this study included ultrapure water (18.2 MΩ cm, Milli-Q), rhodamine 6G (J&K Scientific), N, N-Dimethylformamide (DMF, AR, Aladdin), poly(vinylidene fluoride) (PVDF, M_w=60×10^4, Arkema), Lthium chloride (LiCl, AR, Aladdin), acetone (AR, Sinopharm Chemical Reagent), ethanol (AR, Sinopharm Chemical Reagent), Nafion solution (5wt%, Dupont), commercial IrO_x/Ti mesh catalyst (loading: 0.5 mg/cm^2, Kunshan Shengmolong Electrolytic Equipment), Pt/C (Hipsec4000), carbon paper (Sigracet 29BC), Nafion 115 membrane (N115, Dupont), titanium sheet, aluminum (0.1 mm), alumina ceramic sheets (0.1 mm), ethylene vinyl acetate copolymer (EVA), commercial hydrophilic fabric, acrylic plates.

Assembly of PEM electrolyzer

The PEM electrolyzer was constructed using IrO_x/Ti mesh as the anode, Nafion 115 membrane as the solid polymer electrolyte, Pt/C coated carbon paper as the cathode, and PMMA plate with a serpentine channel as the flow field. The Pt/C cathode was prepared by coating carbon fiber paper with Pt/C catalyst ink (loading: 0.5 mg cm^{-2}), which is composed of a well-dispersed mixture of 20 mg of Pt/C, 3.8 ml of ethanol, and 200 μl of 5 wt% Nafion solution.

Fabrication of the WCL

The WCL was fabricated by electrospinning poly(vinylidene fluoride) (PVDF) nanofibers onto a hydrophilic cotton nonwoven substrate. A 9 wt% PVDF solution was prepared in a DMF/acetone (4:1 v/v) mixture with 0.5 wt% lithium chloride as a spinning aid. The solution was magnetically stirred in a sealed container at 60 °C for 6 hours until fully dissolved, then left to stand until air bubbles dissipated. Prior to electrospinning, the hydrophilic cotton substrate was pre-fixed on a roll. Electrospinning was conducted using a TL-PRO electrospinning apparatus (China) under an applied voltage of 15 kV, a tip-to-collector distance of 15 cm, a flow rate of 0.5 mL h^{-1}, and a 20-gauge needle for 10 hours. The resulting nanofiber layer formed a uniform and porous barrier that facilitates water supply while preventing particulate contamination.

Fabrication of ISVE

The ISVE system included a PV cell, a WCL layer, and a PEM electrolyzer. A self-designed tandem silicon PV cell (16 cm^2) and a Ge/GaInAs/GaInp (12 cm^2) PV cell were used in this work. Aluminum (0.1 mm) or alumina ceramic sheets (0.1 mm) were used as a spacer layer to prevent direct contact between the PV cell and water. The PEM electrolyzer possessed an effective area of 1×1 cm^2. More details can be found in the supplementary document.

Structural and characterization

The X-ray diffraction patterns of the samples were obtained on D8 Discover (Bruker). XPS analyses were performed using a PHI Genesis (Model 500 for XPS/Model 900 for HAXPES). The microscopic structures were characterized by SEM (TESCAN, MIRAS3 FE-SEM). The concentrations of ions in condensates were examined by ICP-OES (PerkinElmer Avio 200). The desorption mass change curves were obtained from the real-time output data of the balance (TPX, 0.1mg in accuracy). The infrared images of the PV cell surface were captured with an infrared camera (Fluke, TiX58). The performance of the PV cells was measured under a solar simulator (AM 1.5 G, ENLITECH, Class AAA). The operating current was recorded by a power meter (66205, Chroma). The solar absorption spectrum was measured by an ultraviolet-visible (UV-vis) near-infrared spectroscope (UV-3600, SHIMADZU) equipped with an integrating sphere (ISR-310). The Farabaric efficiency is analyzed by gas chromatograph (Shimadzu, GC 2014C).

Electrochemical measurements

Electrochemical measurements were executed using a CHI electrochemistry workstation (CHI660, CH Instruments) and a homemade PEM electrolyzer with an active area of 1x1 cm^2. The reactants comprised pumped heated water or in-situ water vapor. Linear sweep voltammetry (LSV) curves were recorded at a scan rate of 10 mV s^{-1}. Electrochemical impedance spectroscopy (EIS) was recorded at 1.6 V.

Tests of evaporative performance

The interfacial evaporation experiment was carried out under a solar simulator. The setup of the evaporation installation is shown in **Fig. S18**. The infrared images of the PV cell under different radiation conditions were captured by an infrared camera. The mass change was recorded in real

time by a high-accuracy balance. In these experiments, the environmental temperature and humidity were maintained at ~25°C and ~40% relative humidity.

Performance evaluation of ISVE and traditional PV-EC devices

For the traditional PV-EC electrolyzer, seawater was fed by a peristaltic pump. For the ISVE electrolyzer, seawater was spontaneously pumped by the WCL through the capillary effect. A solar simulator was used to evaluate the performance of the PV cells. The certified silicon heterojunction cells (KG0 by NERL) were used to calibrate the total intensity to standard solar irradiation. J-V curves were recorded with a scan rate of 10 mV s^{-1}, integrating for 0.01 s and delaying for 0.01 s for each data point. The voltage step was set at 0.02 V. The performance of the solar-driven water/vapor splitting was recorded by a power meter (66205, Chroma). Both ISVE and traditional PV-EC electrolyzer performances were evaluated under simulated solar irradiation (100 mW cm^{-2}, indoor) and natural sunlight (outdoor). The long-term stability test was conducted with photoperiodic lighting (8 hours of light and 16 hours of dark) to simulate night-and-day conditions. The STH efficiency of the long-term stability was normalized relative to the STH value recorded on the first day. The seawater used in this work was collected from the Bohai Sea, China, with an average salinity of ~3 wt %.

The efficiency of STH efficiency was determined using the equation:

$$\mathrm{STH} = \frac{J_{\mathrm{op}} \times E_{\mathrm{f}} \times FE_{\mathrm{H_2}}}{P \times A_{\mathrm{PV}}}$$

Here, J_{op} represents the operating current of the combined system. A_{PV} is the effective illuminated area of the PV cells. E_f is the standard water splitting voltage (1.23 eV), FE_{H_2} is the faradic efficiency for H_2 evolution that is measured to be ～100% and P is the power of solar illumination.

Supplementary Notes

Note 1: Calculation of cost

When calculating the cost of H_2 production for ISVE, we consider the costs of silicon solar cells and all of the components of the electrolyzer: anode, cathode, membrane, as well as a housing component[2]. In this model, we incorporate Ir as the anode catalyst and Pt as the cathode catalyst. Besides, the membrane is considered in between the electrodes to separate the gases and transport protons, and the housing components, including bipolar plates, gas diffusion layers, gaskets, end plates, current collectors, compression bands, stack housing, assembly, and conditioning are also taken into account. PV module and the housing components lifetime are assumed to last 20 years. The catalysts and membrane lifetime are assumed to be 7 years. The discounted rate is 8%[3, 4]. The system can be operated under different current densities or F (A_{EC}/A_{PV}) based on the performance of the silicon solar cells and the electrolyzer (50 °C) in this work (**Fig. S14 and Table S3**). The levelized cost of the H_2 production (LCHP) can be calculated with:

$$LCHP = \frac{\sum_{t=1}^{t=n} \frac{I_t}{(1+r)^t}}{\sum_{t=1}^{t=n} \frac{P}{(1+r)^t}} \tag{2}$$

Where the P is the annual production rate of H_2 (kg cm^{-2}) of PV components which can be estimated from the operating current density of the devices. I_t corresponds to the annual capital cost per cm^2 of PV (\$ cm^{-2}) and it is dependent on the photovoltaic cost and all of the electrolyzer's components costs. The corresponding operating parameters are listed in **Table S4**.

The annual capital cost can be calculated with:

$$I_t = \begin{cases} Cost_{\text{PV}} + F(Cost_{\text{Memb}} + Cost_{\text{Anode}} + Cost_{\text{Cathode}} + Cost_{\text{Housing}}), t = 1 \\ F(Cost_{\text{Memb}} + Cost_{\text{Anode}} + Cost_{\text{Cathode}}), t = 7, 14 \end{cases} \tag{3}$$

The corresponding price parameters of all the components are listed in **Table S5**. The annual production rate of H_2 per cm^2 of PV component is calculated with:

$$P = 365 \times 8 \times \frac{J_{\text{PV}}}{1000} \times \frac{0.5 \text{ mol of H}_2}{96500 \text{ C}} \times \frac{0.002 \text{ kg}}{1 \text{ mol of H}_2} \times 60 \text{ S} \times 60 \text{ min} \tag{4}$$

The calculated results are shown in **Fig. S15**.

Notes S2: Design optimization of ISVE systems based on the electrolyzer/evaporation area to PV cell area ratio (*F*).

When constructing the ISVE, the *F*, defined as the ratio of electrolyzer/evaporation area to PV cell area, is critical, which influence electrolysis efficiency, operating cost and vapor supply. Firstly, to explore the relationship between *F*, temperature, and vapor yield per unit evaporation area, we developed a simplified model using COMSOL Multiphysics. The model simplification process is detailed below:

1. Physics Interfaces: The simulation incorporated heat transfer in moist air and moisture transport in air.
2. Geometry: The silicon solar cell panel was modeled as a rectangular prism with dimensions of 40 mm × 40 mm × 1 mm (length × width × thickness).
3. Boundary Conditions: The top surface of the panel was exposed to solar radiation at an intensity of 1 sun (1 kW/m^2), with a photothermal conversion efficiency of 80%. The top surface also exchanged heat with the environment through thermal radiation (surface emissivity of 0.8) and convective heat transfer with air at an ambient temperature of 25°C. The bottom surface of the panel featured a variable-area evaporation surface. A region 10 mm below the evaporation surface was assumed to be in contact with the environment, maintaining a constant temperature of 25°C and a relative humidity of 40%. All other boundaries were set as thermally insulated to simplify the model.

The simulation results, presented in **Fig. S16**, demonstrate that a lower *F* (ratio of electrolyzer/evaporation area to PV cell area) results in higher operating temperatures, which is beneficial for increasing water electrolysis efficiency (**Fig. 2b**). While a lower *F* also increases the vapor yield per unit evaporation area of the ISVE.

Besides, the *F* also influences the required electrolyzer area, which directly impacts the construction costs of each ISVE unit (**Notes S1**). Typically, a smaller *F* indicates a lower requirement for catalytic area, making a lower *F* more economically appealing.

Moreover, a smaller *F* also means that the electricity generated by the solar cell is applied over a smaller electrolyzer area, as the evaporation and electrolysis areas are the same in our design, thereby increasing the electrolysis current density, thus the vapor demand per unit area.

To quantify this trade-off, we conducted evaporation tests for various *F* values (1, 1/4, 1/9, 1/16, 1/32) to experimentally evaluate the actual vapor supply, and compared these results with the

calculated vapor demand (considering the electroosmotic drag coefficient of 1) under 1 sun illumination. As shown in **Fig. S17**, when F is smaller than 1/16, the vapor supply falls below the vapor demand, making it insufficient to sustain continuous water splitting.

Taken together, these findings indicate that $F = 1/16$ provides the optimal balance—ensuring sufficient vapor availability to meet the electrolysis demand, maintaining high electrolysis efficiency, and minimizing system costs. This integrated consideration of thermal, electrochemical, and economic factors establishes $F = 1/16$ as the most effective design parameter for stable and efficient ISVE operation.

Notes S3: Calculation of the vapor amount needed when coupled with silicon solar cells

For calculating the vapor amount needed of the ideal solar cells under different sunlight intensities, we assumed that all generated electricity is utilized for H_2 production, and the electroosmotic drag coefficient with water vapor is 1[1], which means it takes 3 mol of H_2O to produce 1 mol of H_2. It is assumed that the working current is equal to the short-circuit current of the silicon solar cell under different solar illuminations. Thus, the I_{EC} is ~74, ~149, and ~223 mA cm^{-2} at 0.5, 1, and 1.5 W cm^{-2}, respectively.

Taking the I_{EC} and the electroosmotic drag coefficient into account, the vapor yields (in a unit of g cm^{-2} s^{-1}) needed at the corresponding current density can be calculated with Faraday's law of electrolysis:

$$\dot{m} = \frac{3 \times I_{\mathrm{EC}} \times M_{\mathrm{H_2O}}}{\mathrm{n} \times \mathrm{F}} \tag{1}$$

Where n is the number of electrode reaction charges, and F is the Faraday constant.

Notes S4: Performance prediction of ISVE

The total solar energy absorbed by the ISVE system is split into two parts: electricity and heat. Electricity is utilized for electrolysis, while heat is employed for vapor generation, enabling solar-driven vapor electrolysis for H_2 production. To predict the STH efficiency performance of the ISVE (the F is 1/16), there are some assumptions:

1. The solar energy is first allocated to electrolysis, with η_{PV} varying from 0% to 100%.
2. Subsequently, the remaining energy is dedicated to vapor generation, with η_{hv} ranging from 10% to 100%.
3. The electroosmotic drag coefficient with water vapor is 1[1].
4. Considering that there will be some vapor loss during the actual operation, the vapor utilization rate (φ) is assumed to be 0.8.
5. The faradic efficiency is assumed to be 100% for H_2 evolution.

Here, the I_{EC} related to the PV cells without considering whether the vapor yield amount is sufficient can be calculated with:

$$I_{\mathrm{EC}} = \frac{P \times \eta_{PV}}{F \times E_{\mathrm{f}}} \tag{2}$$

Where I_{EC} (A cm^{-2}) is the current density of the electrolyzer, P (W cm^{-2}) represents solar irradiance, and its value is set at 0.1. F is the area ratio of EC to PV, $A_{\mathrm{EC}}/A_{\mathrm{PV}}$, and the values are set at 1/16.

The I_{vapor} related to vapor yield without considering whether the electricity is enough can be calculated with:

$$I_{\mathrm{vapor}} = \frac{(1-\eta_{PV}) \times P \times \eta_{hv}}{3 \times h \times F \times \mathrm{M_{H_2O}}} \times \mathrm{n} \times \mathrm{F} \tag{3}$$

Where h is the evaporation enthalpy of water at 50 °C and $\mathrm{M_{H_2O}}$ is the molecular weight of water.

Therefore, considering simultaneously adequate electricity and vapor yield for H_2 production, the STH of the ISVE can be calculated with:

$$STH_{\mathrm{ISVE}} = \frac{\mathrm{Min}\{I_{\mathrm{Vapor}}, I_{\mathrm{EC}}\} \times E_{\mathrm{f}} \times F}{P} \tag{4}$$

With equation (8), we plotted the corresponding STH_{ISVE} while varying η_{PV} and η_{hv} in Fig. 5c. When the η_{PV} is less than 50%, surplus solar energy can fully satisfy the vapor amount requirements with η_{hv} of 50%, which can be easily realized via interfacial evaporation[5-7]. However, when the η_{PV} exceeds 50%, a significant portion of the system's energy is dedicated to electricity generation, resulting in insufficient energy for vapor generation. Consequently, the STH efficiency is influenced by η_{hv}, necessitating optimization of the system's η_{hv} to further enhance

the STH efficiency of the ISVE. Fig. S27 shows the STH efficiency of the ISVE as η_{PV} changes, with η_{hv} ranging from 10% to 100%.

Supplementary Figures

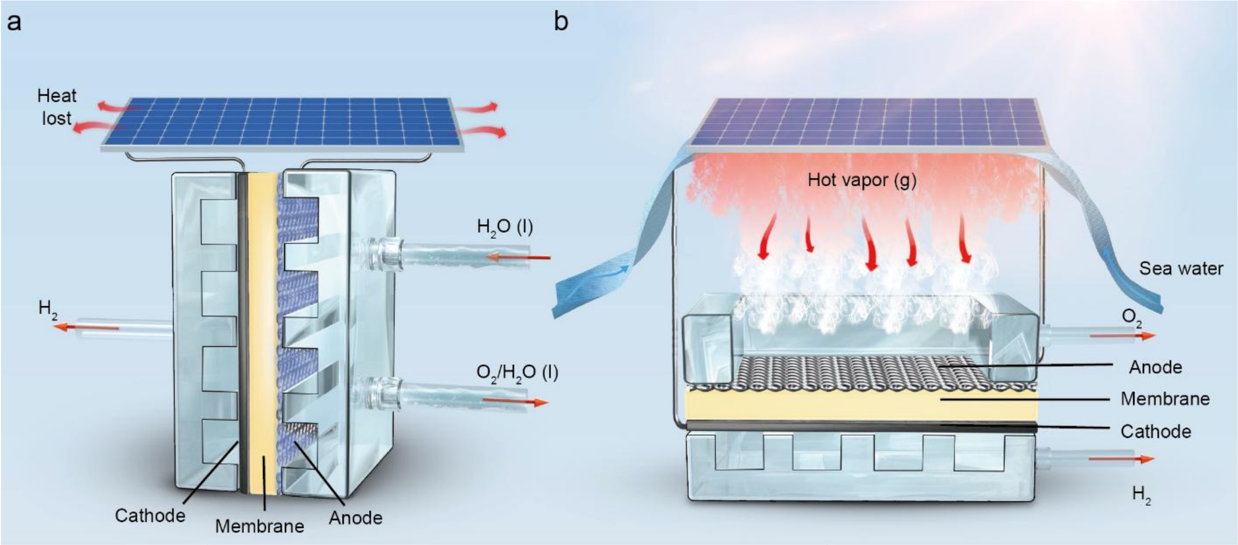

Fig. S1. Schematic of traditional PV-EC electrolyzer (a) and ISVE (b). a, For the traditional PV-EC electrolyzer, including a PV cell and an electrolyzer that are separated spatially, water is fed as the reactant and limits the accessibility of electrochemically active surface sites due to bubble accumulation (Fig. 1d). Besides, the membrane electrode assembly (MEA) electrolyzer requires a high-purity water supply to avoid performance degradation. b, The PV and electrolyzer of ISVE are stacked intimately, and thus the waste heat of solar panels can be utilized to vaporize seawater into high-purity hot vapor for supplying the electrolyzer.

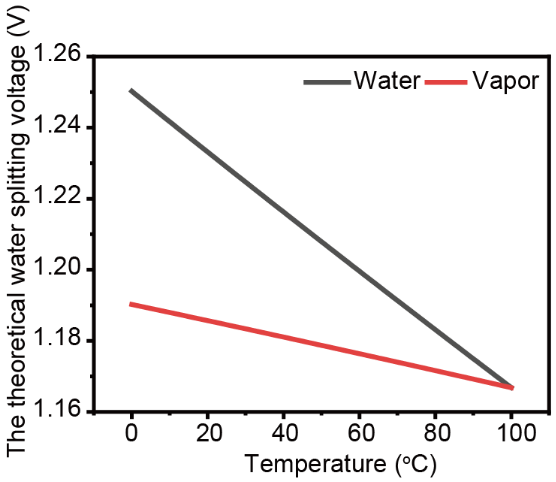

Fig. S2. The comparison of temperature-dependent water splitting voltage with water and vapor as the reactants. For liquid-water and vapor electrolysis, the thermodynamic reversible voltage (E_{RE}) under different temperatures can be calculated using the following equation: $E_{RE} = -\frac{\Delta G}{n\mathrm{F}}$. where n = 2 is the number of transferred electrons for producing 1 mol H_2 and F is the Faraday constant.

Note: The thermodynamic data are adapted from the NIST-JANAF Thermochemical Tables,72, and HSC Chemistry software.

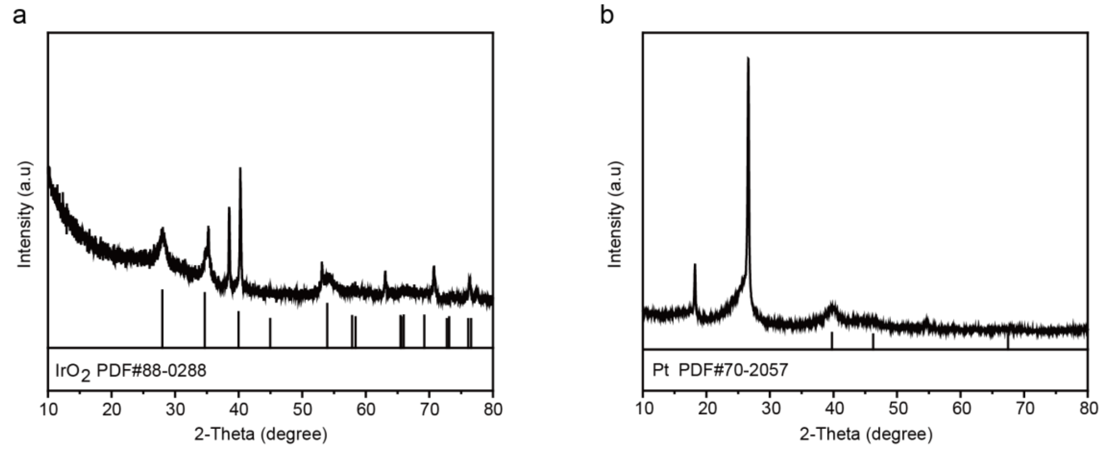

Fig. S3. (a) XRD pattern of the IrO_x/Ti mesh anode. (b) XRD pattern of the Pt/C cathode.

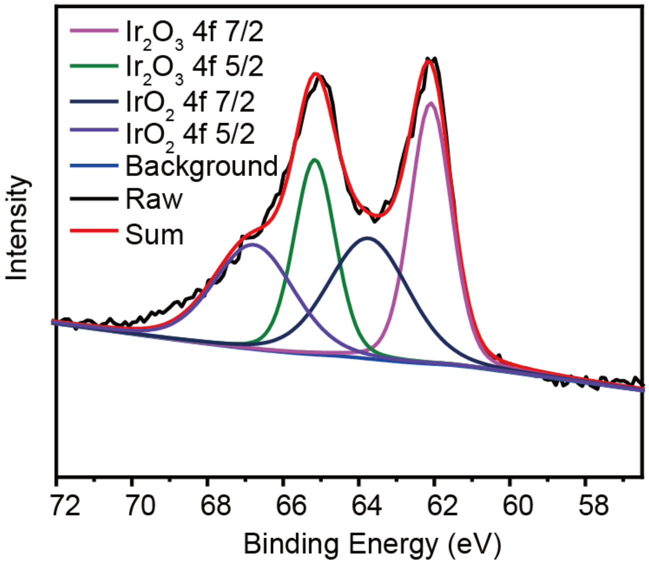

Fig. S4. The deconvolved Ir 4f core-level XPS spectrum of the IrO_x/Ti mesh anode.

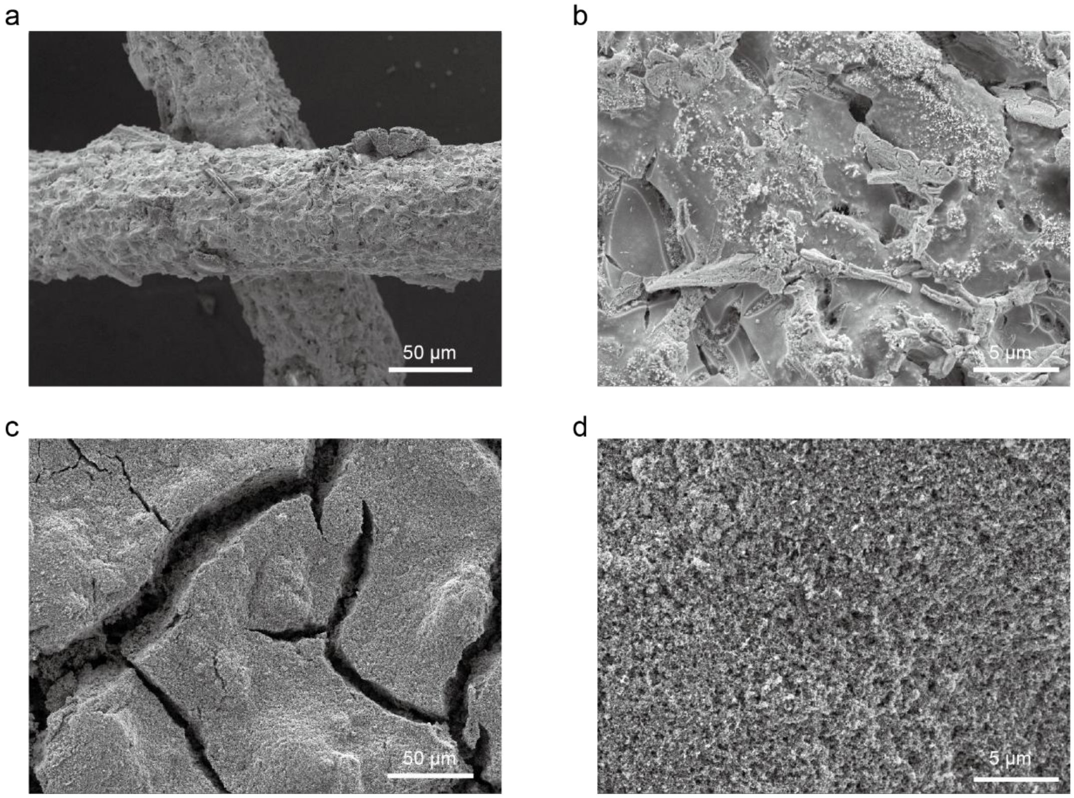

Fig. S5. SEM characterization of the IrO_x/Ti mesh anode (a, b) and Pt/C cathode (c, d).

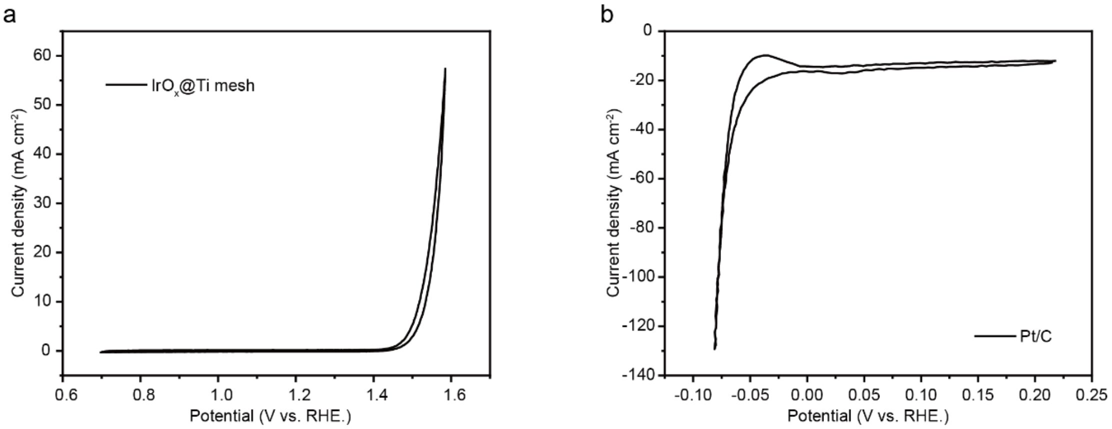

Fig. S6. The recorded CV curves of (a) IrOx@Ti mesh anode and (b) Pt/C cathode in 0.5 M H_2SO_4 electrolyte at the scan rate of 10 mV s^{-1}. The potentials were *iR*-corrected and converted to a reversible hydrogen electrode (RHE) scale.

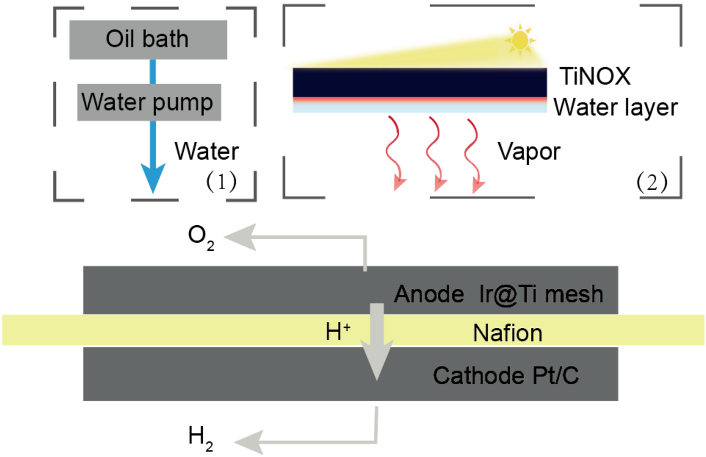

Fig. S7. Schematic diagram of the measuring system for electrolyzer fed with water (1) or vapor (2) at varying temperatures. When fed with water, water is pumped and heated using an oil bath at different preset temperatures. When supplied with vapor, varying temperatures of vapor were generated on the water supply layer using the solar-thermal heat from the solar absorber (TiNOX), and the temperature of vapor depended on the input solar irradiation.

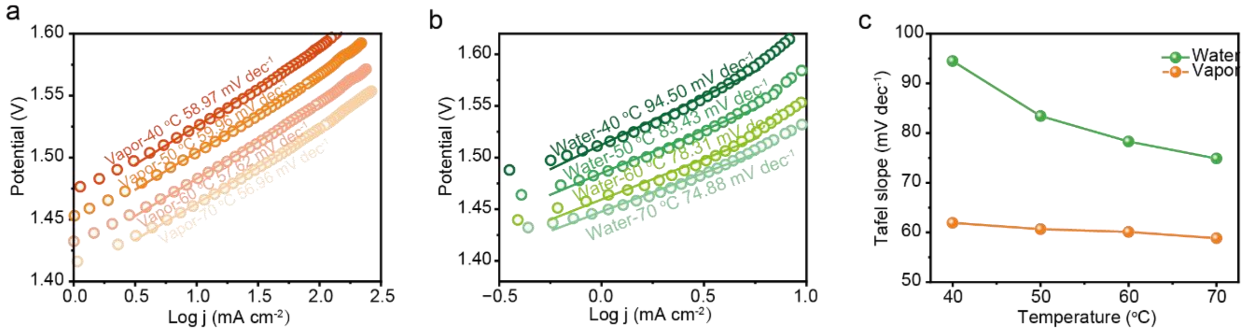

Fig. S8. Derived Tafel slopes of the the (a) vapor-feed electrolyzer and (b) water-feed electrolyzer at different temperatures, along with (c) a summary combining both vapor-feed and liquid-feed data plotted in a single graph.

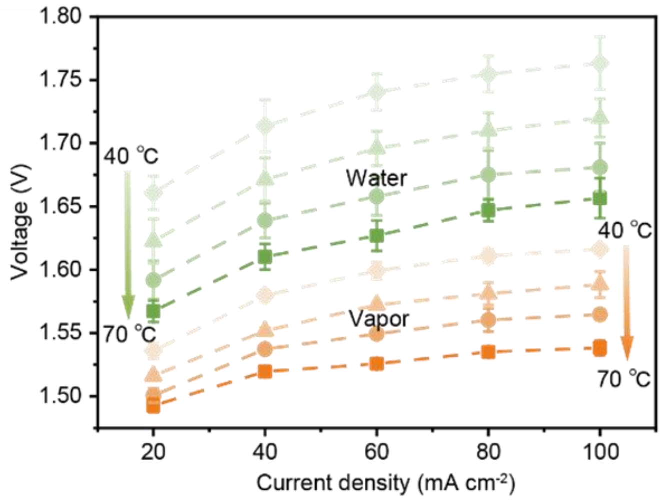

Fig. S9. Applied voltage of two electrolyzers at different temperatures from 20 to 100 mA cm^{-2}. Error bars represent standard errors. Temperature elevation leads to a general reduction in applied voltages for both vapor-fed and liquid water-fed electrolyzers.

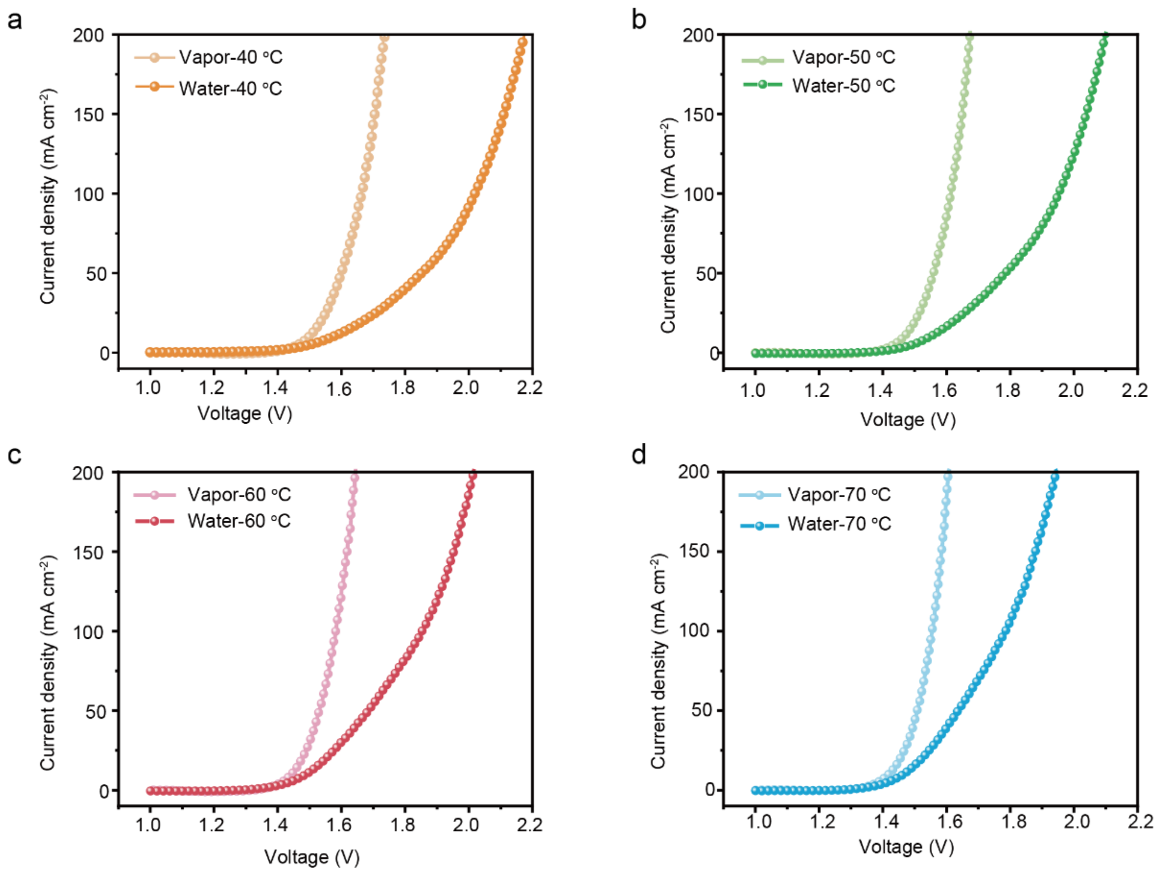

Fig. S10. (a-d) The *iR*-corrected polarization curves of electrolyzers fed with either water or vapor under varied temperatures (40-70 ºC) with RuO_x as the anode catalysts.

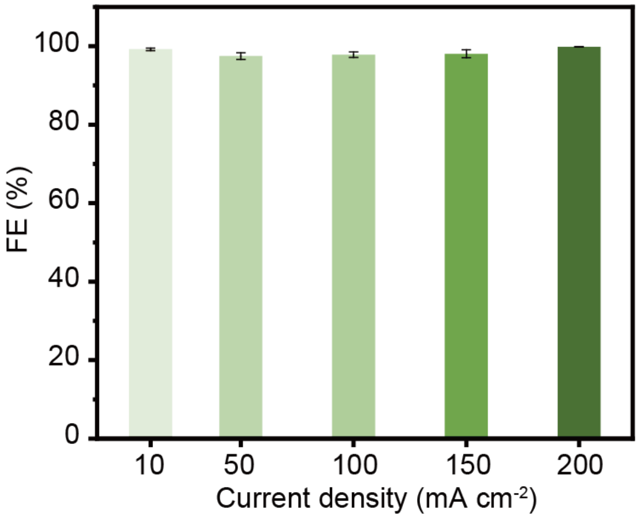

Fig. S11. The Faradaic efficiency (FE) of the PEM electrolyzer in the ISVE system with solar vapor at different current densities. The FE remains close to 100% across the range of current densities, demonstrating the high purity of the vapor generated from seawater, which is crucial for efficient water splitting.

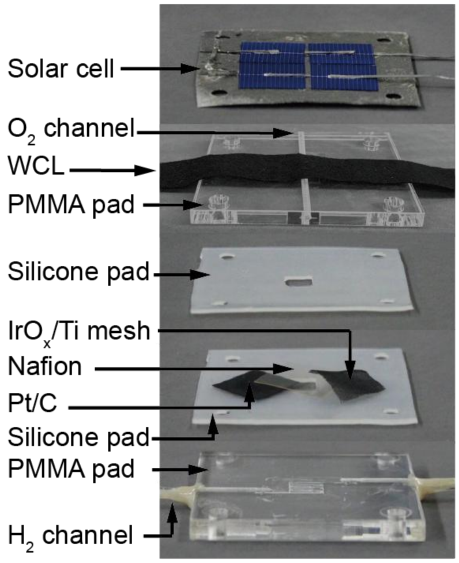

Fig. S12. Components and materials for the ISVE device**.**

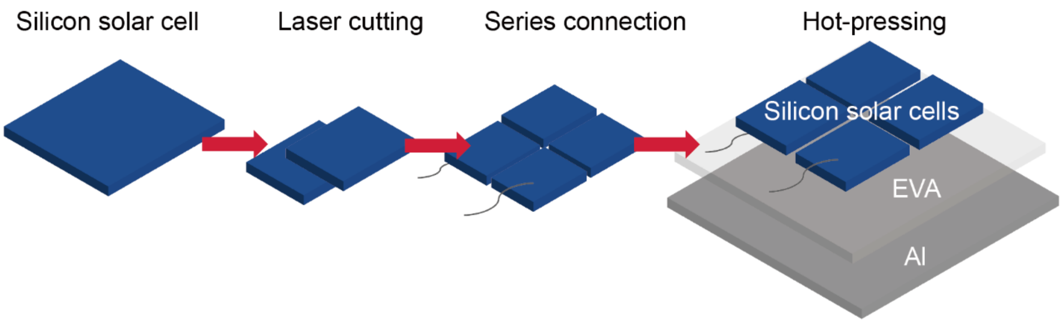

Fig. S13. Scheme of the silicon solar cells preparation. Commercial silicon cells were laser-cut into 2×2 cm² sections using a 1065 nm laser cutting machine (Sunlaite, SLT-PT). These cells were then heated at 110°C for 20 minutes in an oven to minimize edge damage. The cells were then interlinked through the utilization of silver paste and welding tapes, establishing a sequential connection. Following this, the assembled solar cells, along with a 0.4 mm-thick ethylene vinyl acetate (EVA) membrane and a 0.1 mm-thick aluminum (Al) sheet, were effectively enclosed between two Teflon plates. After hot-pressing under 110°C for 10 min in the heating stage, the final configuration of the silicon solar cell was obtained. The incorporation of the aluminum sheet can prevent the solar cells from direct water contact and provide mechanical support and efficient heat transfer.

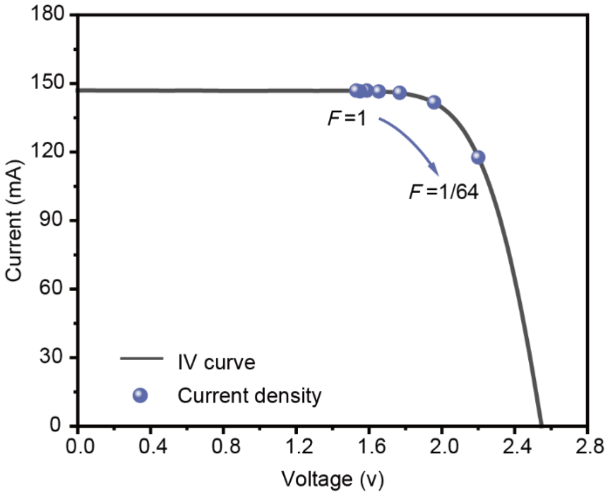

Fig. S14. The operating point of ISVE in the I-V curve of silicon solar cells under different current densities.

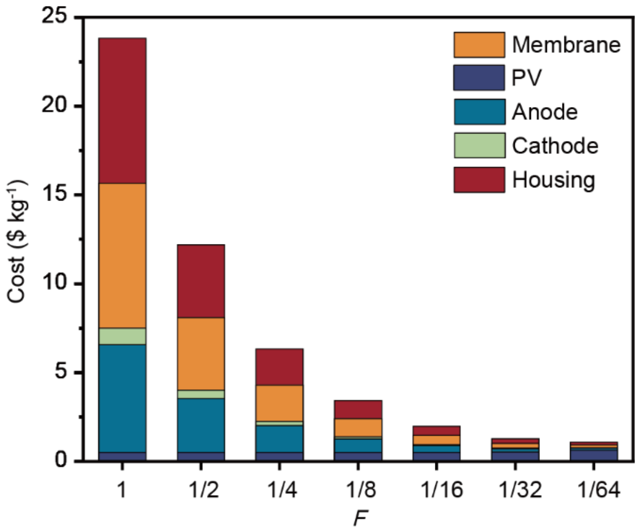

Fig. S15. The relationship between the cost and *F*. The cost contribution for each component is also displayed.

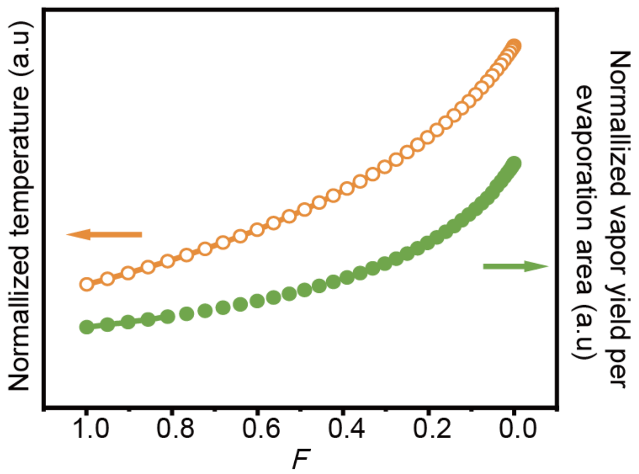

Fig. S16. Normalized simulated temperature and vapor yield per unit evaporation area as a function of factor *F*, which defined as the ratio of electrolyzer/evaporation area to PV cell area. A lower F enhances both temperature and vapor yield per unit evaporation area.

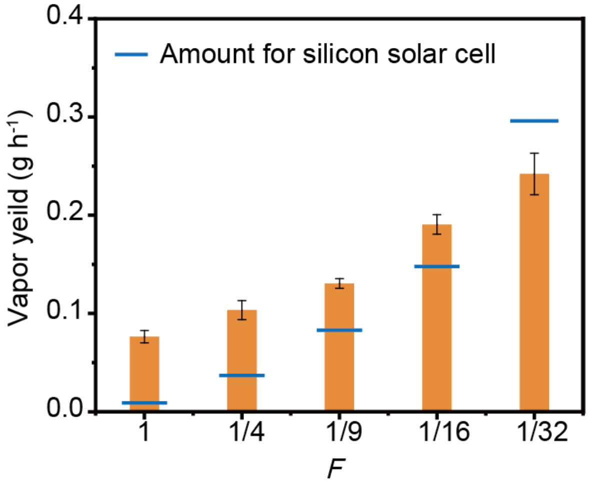

Fig. S17. Vapor yield of ISVE with a silicon PV cell under different *F* value. The required vapor amount for the silicon PV cell is shown as blue lines.

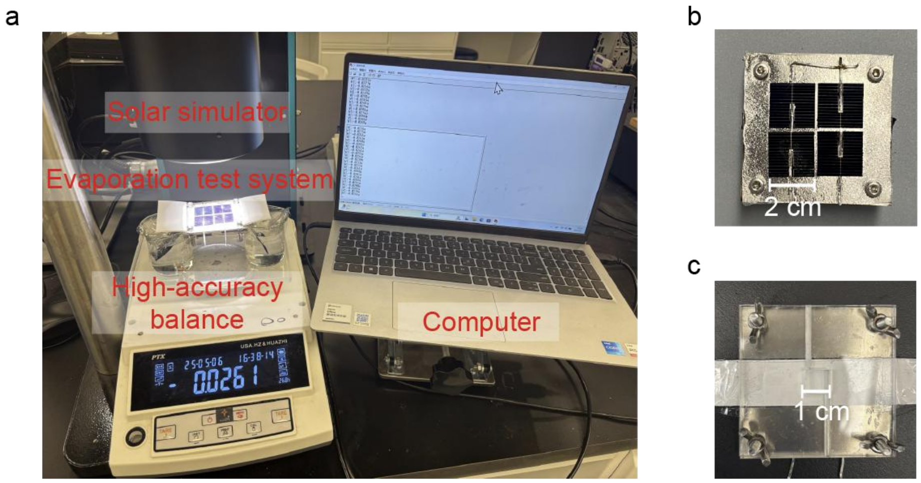

Fig. S18. (a) Optical photo of vapor production performance test system. Front (b) and back (c) views of the silicon solar cell. The solar absorption region of the system corresponds to the area of the silicon solar cell (16 cm^2), while the evaporation area aligns with the catalytically active surface (1 cm^2). Consequently, F, represented by the ratio of evaporation area to solar absorption area, is designed at 1/16 to enhance vapor temperature and reduce catalyst consumption. The vapor yield under different light intensities was tested for 1 hour using seawater. The corresponding vapor yield rate and average temperature recorded by the infrared camera of ISVE with WCL under varying illumination conditions are detailed in Table S2. During three consecutive days for the evaporation test, each day involved 8 hours of evaporation under illumination, generating purified vapor, followed by 16 hours of darkness for salt backflow. A cover seals the evaporation area during dark periods, better simulating nighttime backflow under actual operating conditions.

.

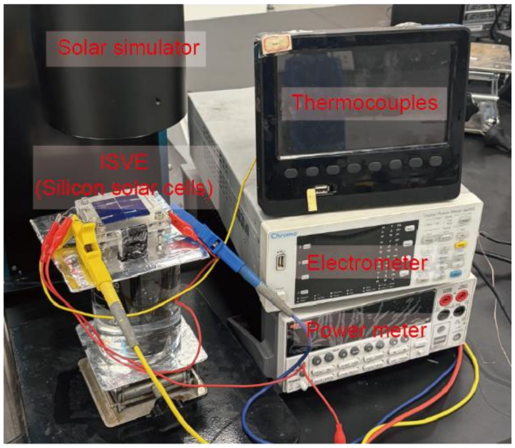

Fig. S19. Optical photo of the ISVE test system. The Keithley DMM6500 electrometer was employed here to evaluate the performance of the solar cell and assess the illumination intensity. Thermocouples were employed for measuring the temperature of the generated vapor, while a power meter was utilized to record the current and voltage across the battery terminals. We tested two different types of solar cells with ISVE. The specific parameters of the solar cells are shown in Table S1. The temperature of the vapor measured from the oxygen channel during the test ranged from 45 to 50 ºC.

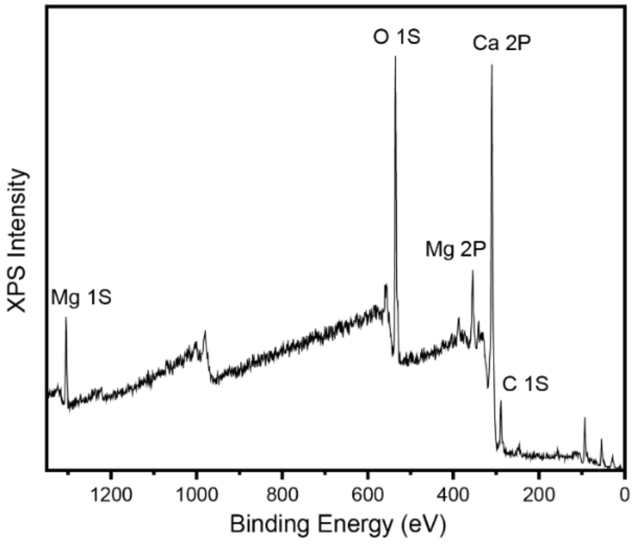

Fig. S20. XPS survey spectrum of the nafion membrane used in seawater feed PV-EC electrolyzer after running for 50 minutes. The result reveals obvious fouling of the membrane following a 50-minute test, characterized by the presence of calcium and magnesium elements. This fouling, attributed to calcium and magnesium, results in a reduction of proton transport capabilities within the membrane. Consequently, this leads to a swift decline in performance within a short timeframe when employing traditional PV-EC systems directly supplied with seawater.

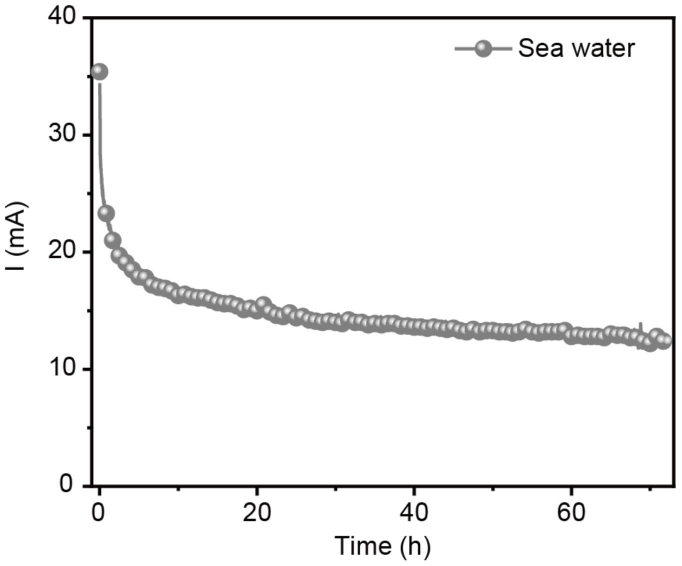

Figure S21. Time-dependent current profile of the traditional PV-EC system fed with seawater.

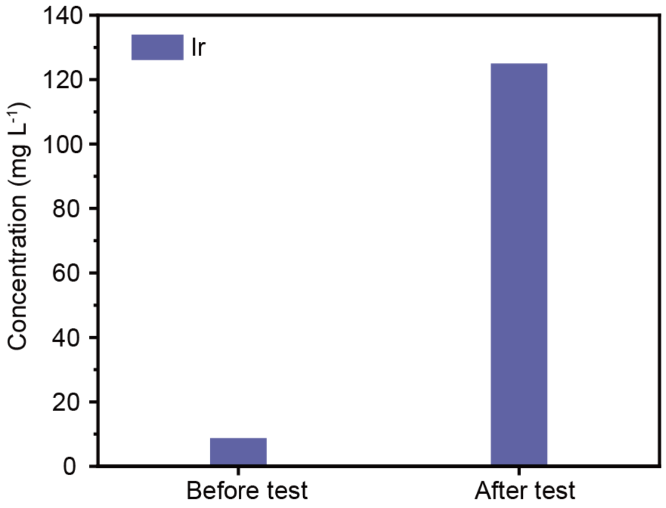

Fig. S22. Concentrations of Ir in seawater anolyte before and after long-term tests in traditional PV-EC. After 72 h of testing, there is a notable increase in the concentration of Ir. This increase can be attributed to the degradation of IrO_x/Ti electrode caused by the Cl-assisted anodization corrosion during the operation.

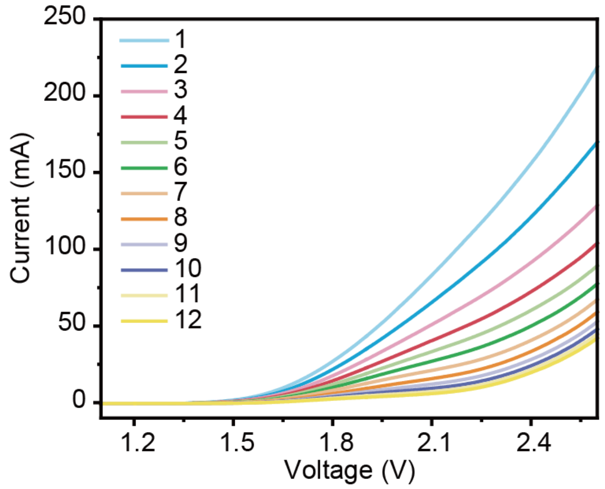

Fig. S23. 12-cycle polarization curve (without *iR*-correction) tests of the seawater-fed PV-EC electrolyzer. The electrochemical performance decreased gradually due to the complex composition of the seawater, causing significant membrane fouling, as well as corrosion or dissolution of the anodic Ir-based catalyst.

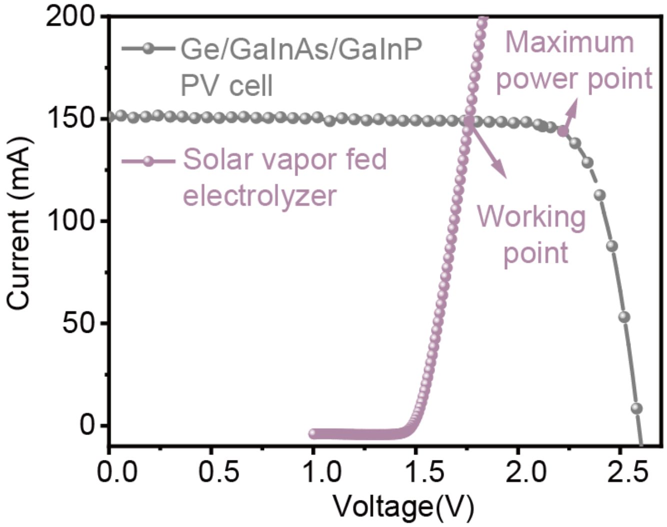

Fig. S24. Polarization curve (without *iR*-correction, effective area: 1 cm^2) of the solar vapor-fed PEM electrolyzer and J-V curve of the Ge/GaInAs/GaInp (12 cm^2) solar cell under simulated one sun illumination (1 kW m^{-2}). The working point is not operating at the maximum power point of the Ge/GaInAs/GaInp solar cell, and the STH efficiency of the ISVE system could be further enhanced with a more suitable solar cell.

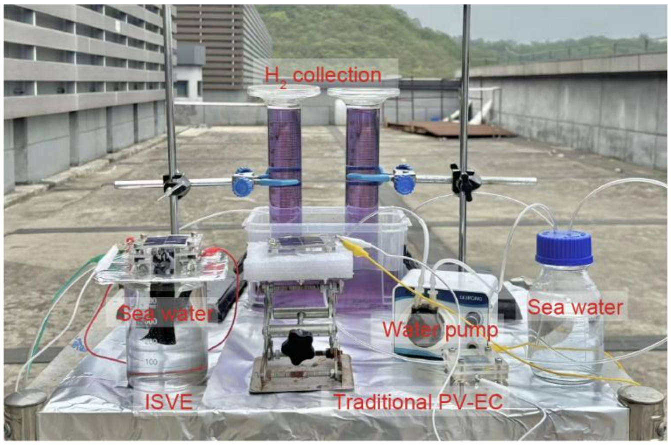

Fig. S25. A photo of the outdoor experimental setup. The outdoor experiment was operated on Nov. 15th, 2022 on the roof of the Zhenjiang building in Nanjing University. In this setup, silicon solar cells with an efficiency of approximately 17% were used for the ISVE system and the traditional PV-EC system. For the reactants, seawater of Bohai was utilized in both systems. In the case of the traditional PV-EC system, seawater was supplied by a water pump. For the ISVE system, seawater was supplied by the fabric-based water layer due to the capillary force. The power meter was employed to record the working current and voltage of the electrolyzers. The H_2 generated at the cathode was collected through a gas channel into an inverted measuring cylinder filled with water. Weather data was systematically recorded by the meteorological station (Tuolaisi, TS-G1), including temperature, solar irradiation, and relative humidity.

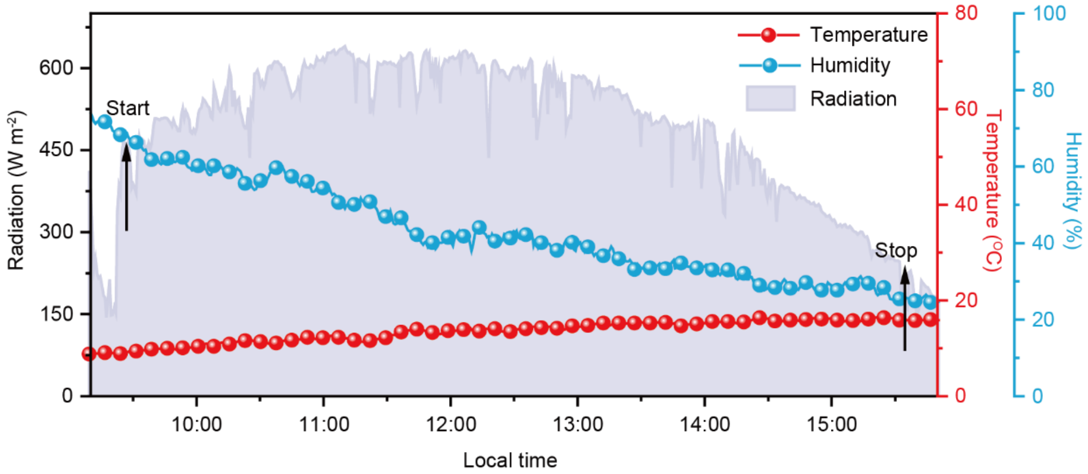

Fig. S26. Temperature, solar irradiation, and relative humidity of the environment. The temperature of the environment during the outdoor experiment ranged from 9 °C to 16 °C, the relative humidity ranged from 25% to 69%, and solar irradiance fluctuated between 180 and 640W m^{-2}.

Environmental factors such as ambient temperature, relative humidity, and solar irradiance jointly influence system performance. Lower temperatures increase heat loss, reducing vapor flux and electrolysis efficiency. However, the enclosed chamber design minimizes the impact of ambient humidity. Since both electricity generation and evaporation rely on the same solar input, changes in irradiance affect both processes proportionally, maintaining internal balance under fluctuating sunlight.

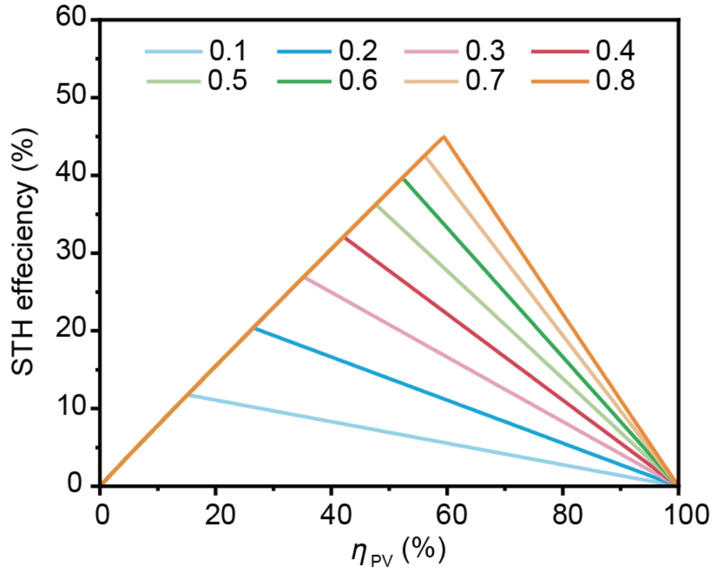

Fig. S27. Detailed simulation results of STH efficiencies for ISVE (the temperature is 50 ºC and the F is 1/16). The PV efficiency (η_{PV}) ranges from 0-100%, with the heat-to-vapor efficiency (η_{hv}) varying from 0.1 to 0.8.

Table S1. Detailed parameters of the solar cells.

	**Silicon PV cell**	**Ge/GaInAs/GaInp PV cell**
J_{sc} (mA cm^{-2})	9.3	12.6
V_{oc} (v)	2.6	2.6
J_{max} (mA cm^{-2})	8.7	12.0
V_{max} (v)	1.94	2.22
Fill Factor (%)	70.8	81.6
Efficiency (%)	16.9	26.6
Area (cm^{2})	16.0	12.0

Table S2. Vapor yield under different illuminations.

Power<br>(kW m^{-2})	Evaporation rate<br>（kg m^{-2} h^{-1}）	Temperature<br>(^{o}C)
0.5	1.31 ± 0.04583	37.3
1	1.91 ±0.10066	45.1
1.5	2.86 ±0.10263	53.7

Table S3. J_{pv} and operating current density of electrolyzer of ISVE under different *F*.

F (A_{EC}/A_{PV})	Operating current density (mA cm^{-2})	J_{pv} (mA cm^{-2})
1/64	470.68	7.35
1/32	283.45	8.86
1/16	145.91	9.12
1/8	73.24	9.16
1/4	36.72	9.18
1/2	18.32	9.16
1	9.18	9.18

Table S4. Operating and financial parameters.

Parameter	Value
Average solar irradiation	1000 W m^{-2}
Total illumination time for 1 year	365 days
Solar irradiation time for 1 day	8 h
System lifetime	20 years
Electrolyzer lifetime	7 years
Discounted rate	8%

Table S5. A detailed summary of the overall costs for the PV-EC system.

	**Value ($ m^{-2})**	**Source**
PV panels	50	[8]
Anode	376	[9]
Membrane	500	[10]
Cathode	53.9	[9]
Membrane housing	500	[2]

Table S6. A summary of STH efficiency, durability, and current density of recent PV-driven water splitting systems.

PV	Water source	PCE (%)	Stability (h)	Peak STH (%)	Last STH (%)	Current (mA cm^{-2})	Source
Self-designed tandem silicon solar cell	Seawater	16.9	1400	11.1 (Average)		144.3	This work
Ge/GaInAs/GaInP, three-junction solar cell	Seawater	26.6	1	15.2 (Average)		147.9	This work
InGaP/GaAs/Ge, a three-junction solar cell	Seawater	32.6	100	17.9	~16.1	14.6	[11]
Perovskite solar cell	Seawater	16	20	11.9	~11.4	9.7	[12]
Commercial solar cell panel	Seawater	24.4	0.58	19.75	~19.75	16.06	[13]
Commercial III-V triple-junction solar cell	Seawater	NA	14	15.13	~15	14.6	[14]
Triple-junction solar cell	Seawater +KOH	NA	NA	12.4		10.1	[15]
Perovskite/Si tandem solar cell	Seawater +KOH	27.42	50	20.61		16.84	[16]
Si	KOH	14.43	90	~10.82		8.89	[17]
Perovskite/Si tandem solar cell	KOH	25.1	2.5	18.0 (Average)		15.2	[18]
Perovskite/Si tandem solar cell	KOH	24.3	15	20	~20	16.27	[8]

Perovskite tandem solar cell	NaOH	15.7	2	12.3	9	9.61	[19]
Crystalline Si solar cell	Alkali solution	24.3	50	16.9 (Average)		51	[20]
3 interconnected silicon HJ	KOH	20.6	100	14.5	14.2	11.8	[21]
AsGa solar cells	KOH	NA	NA	18.1		14.7	[22]
Perovskite tandem cell	KOH	14.69	2	~11.22		9.12	[23]
Perovskite tandem cell	KOH	16.8	20	13	11.31	11.06	[24]

Table R7. A summary of STH efficiency, durability, and current density of recent PEC water splitting systems.

Water source	Stability (h)	Peak STH (%)	Last STH (%)	Current (mA cm^{-2})	Source
0.5 M phosphate buffer	10	~0.91		0.8	[25]
0.5 M KPi + 0.01 M V_2O_5	10	1~.5		4.7	[26]
1.0 M K_2SO_4	1	~2.1%		2.1	[27]
0.2 M K-borate buffer	12	3	2.7	2.45	[28]
0.1 M KB+ 0.1 M K_2SO_4	96	1.26	~0.9	15	[29]
0.2 mol dm^{-3} Na_2HPO_4/NaH_2PO_4 ,	60	3.17		2.58	[30]
1.0 M PBS	12	~5.21		4.3	[31]
0.5 M K-Pi	10	6.75		5.49	[32]
1.0 M NaOH	20	~5.03		11.84	[33]
0.5 M H_2SO_4	200(min)	~4.3		11.98	[34]
0.5 M H_2SO_4 + 0.1 M KI	2	4.9	1.5	~4	[35]

Table S8. Summary of η_{hv} for water and electricity cogeneration systems.

η_{solar}	η_{pv}	$\eta_{evaporation}$	η_{hv}	Source
74.6	20.4	54.2	68.1	[5]
88.8	12.2	76.6	87.2	[6]
78.3	17.4	62.2	75.2	[7]

Note: η_{solar} is an overall solar energy utilization efficiency of 1sun illumination, and $\eta_{evaporation}$ is defined as $\dot{m}h/P$, where $\dot{m}$ is the mass flux of vapor, h is the liquid-vapor phase change enthalpy and P is the solar energy.

References

1. Zawodzinski, T.A., et al., The water content dependence of electro-osmotic drag in proton-conducting polymer electrolytes. *Electrochimica acta* 1995; **40**: 297-302.
2. Rodriguez, C.A., et al., Design and cost considerations for practical solar-hydrogen generators. *Energy Environ. Sci.* 2014; **7**: 3828-3835.
3. Rajeshwar, K., R.D. McConnell, and S. Licht, Solar hydrogen generation: toward a renewable energy future. *(Springer.(New York),2008)*
4. Coppitters, D., W. De Paepe, and F. Contino, Surrogate-assisted robust design optimization and global sensitivity analysis of a directly coupled photovoltaic-electrolyzer system under techno-economic uncertainty. *Appl. Energy* 2019; **248**: 310-320.
5. Xu, N., et al., Synergistic tandem solar electricity-water generators. *Joule* 2020; **4**: 347-358.
6. Ji, Q., et al., Synergistic solar-powered water-electricity generation via rational integration of semitransparent photovoltaics and interfacial steam generators. *J Mater Chem A* 2021; **9**: 21197-21208.
7. Liu, M., et al., Synergistic solar-powered water-electricity generation: an integrated floating system on water. *Nano Energy* 2024; **119**: 109074.
8. Wang, Y., et al., Direct solar hydrogen generation at 20% efficiency using low-cost materials. *Adv Energy Mater* 2021; **11**: 2101053.
9. *https://pmm.umicore.com/en/prices*.
10. Mayyas, A.T., et al., *Manufacturing cost analysis for proton exchange membrane water electrolyzers*. (National Renewable Energy Lab.(NREL), Golden, CO (United States), 2019).
11. Hsu, S.H., et al., An earth-abundant catalyst-based seawater photoelectrolysis system with 17.9% solar-to-hydrogen efficiency. *Adv Mater* 2018; **30**: 1707261.
12. Kuang, Y., et al., Solar-driven, highly sustained splitting of seawater into hydrogen and oxygen fuels. *Proc Natl Acad Sci* 2019; **116**: 6624-6629.
13. Chang, K., et al., Atomic heterointerface engineering of Ni2P-NiSe2 nanosheets coupled ZnP-based arrays for high-efficiency solar-assisted water splitting. *Adv Funct Mater* 2022; **32**: 2113224.
14. Wang, C., et al., Heterogeneous bimetallic sulfides based seawater electrolysis towards stable industrial-level large current density. *Appl. Catal., B* 2021; **291**: 120071.
15. Sun, J., et al., Joule heating synthesis of well lattice-matched Co2Mo3O8/MoO2 heterointerfaces with greatly improved hydrogen evolution reaction in alkaline seawater electrolysis with 12.4% STH efficiency. *Appl. Catal., B* 2023; **338**: 123015.
16. Pan, S., et al., Floating seawater splitting device based on NiFeCrMo metal hydroxide electrocatalyst and perovskite/silicon tandem solar cells. *ACS nano* 2023; **17**: 4539-4550.
17. Riyajuddin, S., et al., Super-hydrophilic leaflike Sn4P3 on the porous seamless graphene–carbon nanotube heterostructure as an efficient electrocatalyst for solar-driven overall water splitting. *ACS nano* 2022; **16**: 4861-4875.
18. Gao, J., et al., Solar water splitting with perovskite/silicon tandem cell and TiC-supported Pt nanocluster electrocatalyst. *Joule* 2019; **3**: 2930-2941.
19. Luo, J., et al., Water photolysis at 12.3% efficiency via perovskite photovoltaics and Earth-abundant catalysts. *Science* 2014; **345**: 1593-1596.
20. Chen, H., et al., Co and Fe Codoped WO2. 72 as Alkaline-Solution-Available Oxygen Evolution Reaction Catalyst to Construct Photovoltaic Water Splitting System with Solar-To-Hydrogen Efficiency of 16.9%. *Adv. Sci.* 2019; **6**: 1900465.
21. Schüttauf, J.-W., et al., Solar-to-hydrogen production at 14.2% efficiency with silicon photovoltaics and earth-abundant electrocatalysts. *Journal of The Electrochemical Society* 2016; **163**: F1177.
22. Sun, P., et al., Round-the-clock bifunctional honeycomb-like nitrogen-doped carbon-decorated Co2P/Mo2C-heterojunction electrocatalyst for direct water splitting with 18.1% STH efficiency. *Appl. Catal., B* 2022; **310**: 121354.

23. Weng, B., et al., A layered Na 1− x Ni y Fe 1− y O 2 double oxide oxygen evolution reaction electrocatalyst for highly efficient water-splitting. *Energy Environ. Sci.* 2017; **10**: 121-128.
24. Wang, M., et al., Heterostructured Co/Mo-sulfide catalyst enables unbiased solar water splitting by integration with perovskite solar cells. *Appl. Catal., B* 2022; **309**: 121272.
25. Jang, J.-W., et al., Enabling unassisted solar water splitting by iron oxide and silicon. *Nat. commun.* 2015; **6**: 7447.
26. Yang, W., et al., Benchmark performance of low-cost Sb2Se3 photocathodes for unassisted solar overall water splitting. *Nat. commun.* 2020; **11**: 861.
27. Vijselaar, W., et al., Spatial decoupling of light absorption and catalytic activity of Ni–Mo-loaded high-aspect-ratio silicon microwire photocathodes. *Nat. Energy* 2018; **3**: 185-192.
28. Pan, L., et al., Boosting the performance of Cu2O photocathodes for unassisted solar water splitting devices. *Nat. Catal.* 2018; **1**: 412-420.
29. Pornrungroj, C., et al., Bifunctional perovskite-BiVO4 tandem devices for uninterrupted solar and electrocatalytic water splitting cycles. *Adv Funct Mater* 2021; **31**: 2008182.
30. Huang, D., et al., 3.17% efficient Cu 2 ZnSnS 4–BiVO 4 integrated tandem cell for standalone overall solar water splitting. *Energy & Environmental Science* 2021; **14**: 1480-1489.
31. Wang, L., et al., A transparent, high-performance, and stable Sb2S3 photoanode enabled by heterojunction engineering with conjugated polycarbazole frameworks for unbiased photoelectrochemical overall water splitting devices. *Adv. Mater.* 2022; **34**: 2200723.
32. Huang, J., et al., Highly efficient sustainable strategies toward carbon-neutral energy production. *Energy Environ. Sci.* 2024; **17**: 1007-1045.
33. Cai, Y., et al., Semi-transparent and stable In2S3/CdTe heterojunction photoanodes for unbiased photoelectrochemical water splitting. *Nat. Commun.* 2025; **16**: 5105.
34. Ye, S., et al., Unassisted photoelectrochemical cell with multimediator modulation for solar water splitting exceeding 4% solar-to-hydrogen efficiency. *J. Am. Chem. Soc.* 2021; **143**: 12499-12508.
35. Park, Y.S., et al., High-performance Sb 2 S 3 photoanode enabling iodide oxidation reaction for unbiased photoelectrochemical solar fuel production. *Energy Environ. Sci.* 2022; **15**: 4725-4737.
